# Golodirsen restores DMD transcript imbalance in Duchenne Muscular Dystrophy patient muscle cells

**DOI:** 10.1186/s13395-024-00360-4

**Published:** 2024-11-29

**Authors:** Rachele Rossi, Silvia Torelli, Marc Moore, Pierpaolo Ala, Jennifer Morgan, Jyoti Malhotra, Francesco Muntoni

**Affiliations:** 1 The Dubowitz Neuromuscular Centre, UCL Great Ormond Street Institute of Child Health, London, UK; 2 Great Ormond Street Institute of Child Health Biomedical Research Centre, National Institute for Health Research, University College London, London, UK; 3 Sarepta Therapeutics, Cambridge, MA USA; 4 National Horizons Centre, Teesside University, Darlington, DL1 1HG UK

**Keywords:** Antisense oligonucleotide, Duchenne muscular dystrophy, Golodirsen, Morpholino, Transcript imbalance.

## Abstract

**Background:**

Antisense oligonucleotides (AON) represent a promising treatment for Duchenne muscular dystrophy (DMD) carrying out-of-frame deletions, but also show limitations. In a completed clinical trial golodirsen, approved by FDA to induce skipping of DMD gene exon 53 in eligible patients, we demonstrated increase in DMD expression and protein production, albeit with inter-patient variability.

**Methods:**

Here, we investigate further the golodirsen mechanism of action using myotubes derived from MyoD transfected fibroblasts isolated from DMD patients at the baseline of the clinical trial SRP-4053.

**Results:**

We confirm golodirsen’s selectivity and efficiency in removing only exon 53. For the first time in human cells, we revealed a significant reduction in the so called DMD “transcript imbalance”, in golodirsen-treated DMD muscle cultures. The transcript imbalance is a unique DMD phenomenon characterized by non-homogeneous transcript expression along its entire length and responsible for the reduced stability of the transcript. Our in-vivo study also showed that the efficiency of exon skipping did not always correspond to a proportional restoration of the dystrophin protein. Predominant nuclear localization of the DMD transcript, observed in patients and animal models, persists even after exon skipping.

**Conclusion:**

All these findings suggest challenges other than AON delivery for high level of protein restoration in DMD, highlighting the importance of investigating the biological mechanisms upstream of protein production to further enhance the efficiency of any AON treatment in this condition.

**Supplementary Information:**

The online version contains supplementary material available at 10.1186/s13395-024-00360-4.

## Background

Duchenne muscular dystrophy (DMD) is a severe, X-linked recessive disorder with an incidence of 1 in 3,500 newborn males worldwide [1]. The disease is characterized by progressive loss of muscle mass and function, muscle weakness, leading to a loss of ambulation by the early teenage years. Respiratory complications and dilated cardiomyopathy significantly affect the patient’s survival [2, 3] and premature death commonly occurs between the 3rd and 4th decade of life [4]. Becker muscular dystrophy (BMD) is a milder allelic form characterized by a slower disease progression than DMD, less severe muscle weakness, atrophy and with ambulation retained into adulthood in most patients [5]. DMD is caused by mutations in one of the largest known human genes, the *DMD* gene, spanning 2.5 Mb, that consists of 79 exons and that codes for 7 tissue specific transcripts: 3 long and 4 short DMD isoforms [6]. The majority of DMD mutations are gross rearrangements, especially deletions, which have a range of frequency, variable among populations, between 57 and 80%. Large duplications instead are less frequent ranging between 5 and 11%. The remaining variations are small mutations and intronic copy number variations (CNVs) [7]. Generally, 1 out of 3 are de novo mutations and, even though they can occur throughout the entire length of the gene, there is a recognised deletion cluster encompassing exons 45–55 and a duplication hotspot between exon 2 to 10 [8, 9]. The majority of pathogenic *DMD* mutations interrupt the translational open reading frame causing the almost complete absence of dystrophin protein, resulting in the severe DMD clinical phenotype; whereas mutations that preserve the reading frame permit a shorter but functional protein product linked to the milder BMD phenotype [10]. Antisense oligonucleotides (AONs) are small, synthetic nucleic acids complementary to their target pre-mRNA, and they represent a promising genetic therapy approach to treat DMD. AONs are indeed able to restore the *DMD* reading frame by removing a neighbouring out-of-frame exon, allowing the production of a shortened but functional dystrophin protein mimicking the protein produced by patients with BMD [11]. Golodirsen, formerly SRP-4053 and commercially known as Vyondys 53™, is a phosphorodiamidate morpholino oligomer (PMO) that specifically targets the *DMD* exon 53. Together with eteplirsen and casimersen, it is an FDA approved gene therapy for Duchenne, commercialised by Sarepta Therapeutics. The fourth FDA-approved antisense for the treatment of DMD is viltolarsen developed by NS Pharma Inc which also skips the exon 53 of the *DMD* gene [12]. All of these AON PMOs show a modest but consistent increase of dystrophin production in biopsies collected after treatment compared to baseline biopsies [13, 14]. Multiple reasons can be responsible for the limited amount of protein restoration, including the insufficient AONs cellular uptake, a common phenomenon for AON therapies, with less than 1% of first generation AONs reaching the correct cellular compartment [12]. To address this limitation, several companies are working on the AON chemical structure by conjugating various peptides to PMOs to increase their cellular uptake, such as the peptide-morpholino oligomer conjugates (PPMOs) or exploit receptor mediated uptake using transferrin-receptor conjugated AONs [12].

On the other hand, recent findings suggest that some of the mutant *DMD* transcript’s peculiarities could play a crucial role in limiting the effectiveness of AONs. Indeed, recent reports have underlined the lower level of *DMD* transcript present in patients compared to healthy controls [15], meaning less substrate available in all those therapies having mRNA as their biological target. Furthermore, DMD patients appear to have a high rate of transcript imbalance, which consists in the *DMD* transcript not being homogeneously expressed throughout its length, with the 5’end more expressed than the 3’end. Even though the *DMD* transcript imbalance was described for the first time in 1995 [16] and it is relevant for disease pathophysiology [17], the repercussions for mRNA-based therapies have been addressed only in animal models [18]. The wider implications of this phenomenon for DMD therapies are therefore still poorly understood. Another relevant aspect only recently investigated is intracellular trafficking of mutant transcripts, that appears to be compromised in DMD patient’s cells, *mdx* mouse and canine models versus healthy controls and wild type (WT) animals [17, 19, 20]. In-vivo and in-vitro studies in dystrophic *mdx* mouse indeed suggest that the *Dmd* transcript may have a nuclear preferential localization which may not be influenced by AON treatment, hence limiting the availability of this transcript in the cytoplasm [17]. This paper aims to provide an in-depth analysis of golodirsen’s mechanism of action and the biological factors that directly affect the activities of AONs in patients affected by DMD.

In this paper, golodirsen’s mechanism of action was investigated by comparing treated with non-treated differentiated MyoD lentivirally - transfected fibroblasts [21, 22] from 25 DMD patients previously recruited into a phase I/II trial on golodirsen [23]. Both exon 53 skipping and protein restoration percentages were measured to evaluate golodirsen’s efficiency. Next, the specificity and the mechanism of action of golodirsen was investigated by analysing the expression of all exon-exon junctions through the *DMD* transcript in all patients by FluiDMD cards, confirming that golodirsen selectively removed only exon 53 from the transcript; the analysis did not find any unspecific skipping product or aberrant transcript in the treated cultures, confirming the fidelity of the target engagement. The evaluation was extended to include the *DMD* transcript stability and its sub-cellular localization to study whether these aspects could be positively influenced by the therapeutically restored reading frame. Finally, in order to have a complete overview of our patient cohort and assess more deeply the effect of mutations on exon skipping efficiency, the patients’ genome was sequenced, and the deletion breakpoints annotated and analysed for the repetitive elements in their surroundings.

## Results

### Golodirsen induced exon 53 skipping at high efficiency and increased DMD transcript in patient myogenic cultures

**Fig. 1:**
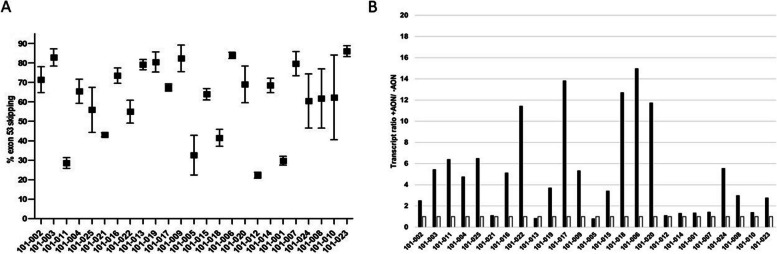
Exon 53 skipping efficiency & transcript amount. **A** Graphical representation of exon skipping efficiency percentage (Y-axis) among patients (X-axis). Squares represent the mean exon skipping percentage for 3 technical replicates per sample ± SD. **B **Graphical representation of *DMD* transcript expression in treated cultures (black bars) normalised to non-treated cultures (white bars). Non-treated transcripts amount among samples are set as 1 to facilitate the graphical visualization. Transcript expression (Y-axis) is expressed as ratio between treated and non-treated cultures

 Agarose gel electrophoresis was used as first analysis to visualize the existence of the new exon-exon junction generated after exon 53 skipping-AON mediated (Supplementary Figure S1) and to confirm the previous finding observed in the in-vitro study on the same patients’ muscle biopsies [23]. The percentage of *DMD* exon 53 skipping was successively evaluated, in treated versus non-treated cells, in a paired way, for each patient by Taqman RT-qPCR. We found that golodirsen promoted exon skipping in all 25 treated patients’ cells with a range of efficiency between 13 and 86.16% and an average of 54.50% (Fig. 1A).

Differences in skipping efficiency average and standard deviation were observed among deletion types (Table S1) but these did not reach significance. In accordance with previously described findings [24–26], we observed a critical reduction or absent AON mediated exon skipping in healthy controls (HCs). Indeed, exon skipping evaluation conducted on 5 golodirsen-treated HCs (3 biological replicates and 2 technical replicates) ranged from 0 to 1.53%, with an average of 0.42% (Table S2), highlighting significant and substantial differences between DMD and healthy control myogenic cells in PMO antisense action and/or delivery properties.

The total transcript amount, for the full length *DMD* Dp427 isoform, was evaluated using the expression of the *DMD* exon-exon junction 55–56. This specific exon-exon junction was selected to be close to golodirsen’s site of action and avoiding inaccuracies in the estimation of transcript expression or exon skipping efficiency, due to the transcript imbalance influence.

Most of the treated patient cultures (92%) showed an average increment of *DMD* transcript amount when compared to non-treated samples (Fig. 1B) with only two patients demonstrating unchanged expression levels of *DMD* transcript between pre and post golodirsen administration.

These data indicate that golodirsen was able to significantly (two tailed *P*-val < 0.0001) increase the amount of *DMD* transcript in cells with a range of expression from 0.8 to 14.9-fold (Table S3) between treated and non-treated DMD cell cultures. The significant increment of transcript after golodirsen treatment was demonstrated further by testing individual replicates of treated versus non treated samples (Supplementary figure S2, two tailed *P*-val < 0.00001) and observing that 85.3% of treated replicates show a *DMD* transcript increment.

#### Golodirsen significantly restores the *DMD* transcript imbalance and does not promote unspecific *DMD* skipping events

**Fig. 2:**
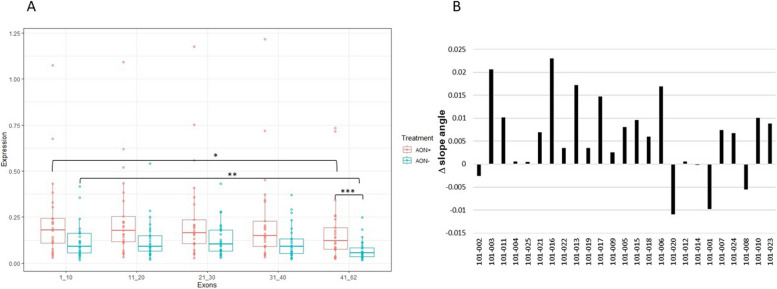
5’−3’ *DMD* transcript imbalance visualization and its restoration after golodirsen treatment. **A** Boxplot with individual data points offer the visual representation of the *DMD* transcript imbalance, through transcript length (5’−3’). Transcripts’ expression, calculated by the 2^-DDCt methods from FluiDMD cards data, (Y-axis) were plotted against the average of around 10 exon-exon junctions (X-axis) until the exon-exon junction ex61-ex62, to avoid the Dp71 isoform contribution. Dots indicate values for each individual data point, representing the single patient contribution for each interval of exon-exon junctions. 5’ junctions are higher expressed than 3’ junctions in both treated (pink boxes) and non-treated (blue boxes) groups. Exon-exon junctions surrounding the deletions and downstream the exon 62 were removed to avoid underestimation and the Dp71 contribution, respectively. (Wilcoxon test *p*-value, *= *p* < 0.001, **= *p* < 0.001, ***= *p* = 0.003. **B** Transcript imbalance restoration (Y-axis), after golodirsen administration, was evaluated by subtracting from the slope angle value of the treated sample (+ AON) the non-treated sample (-AON) for each patient (X-axis). Only 5 patients did not show any reduction in transcript imbalance after golodirsen (negative bars)

All the exon-exon junctions, through the *DMD* transcript length, were evaluated by FluiDMD cards to observe the *DMD* transcript’s expression trend (the so-called transcript imbalance). Even though the *DMD* transcript was more highly expressed in treated compared to non-treated cultures, in all patients the exon-exon junction groups showed a significantly decreasing level of expression from the transcript 5’-end to its 3’end (for both treated * and non-treated ** cultures the Wilcoxon test *p*-value is < 0.001). However, this trend was less pronounced in the treated patients (Fig. 2A pink boxes), which have a significantly higher median expression of the exons 41–62 compared to the non-treated samples (Fig. 2A blue boxes) (*** Fig. 2A, Wilcoxon test *p*-value = 0.003). To verify, and precisely quantify, the transcript imbalance reduction or the transcript imbalance restoration, the imbalance rates of non-treated (-AON) samples were subtracted to all treated (+ AON) samples. Slope angle of regression lines, drawn among the cycles threshold (Cts) relative to all exon-exon junctions and plotted on the X-axis of a histogram graph (Figure S3), was used to assess the transcript imbalance rates (Table S4) for each patient’s cells. The results showed that golodirsen treatment modestly but significantly (Wilcoxon test, 95% of confidence, two tailed *P*-val < 0.0001) restored the imbalance rate in 80% of treated samples (Fig. 2B).

Similarly, we also compared + AON samples with HCs cultures to test the rate of transcript imbalance recovery. We found that despite the exon skipping-golodirsen mediated induced restoration of the reading frame, the improvement was not able to completely recover the transcript imbalance in patients to levels comparable to healthy controls (Figure S4).

### Restored dystrophin protein has the expected molecular weight and its quantity is significantly higher after golodirsen treatment than in non-treated cultures

**Fig. 3:**
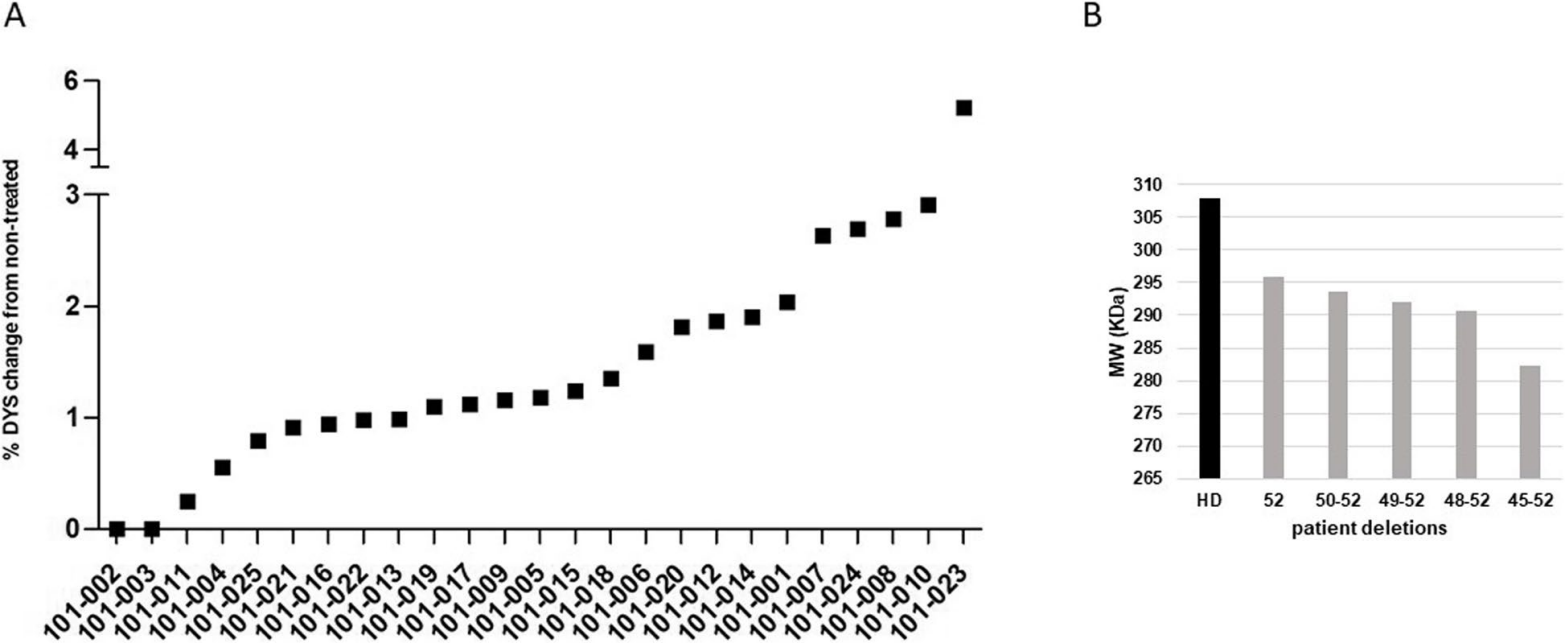
Dystrophin protein restoration after treatment and its average size.** A** Graphical representation of DYS restoration in treated samples expressed as % of dystrophin change from non-treated (Y-axis) samples among patients (X-axis). All non-treated patient cells show no expression of dystrophin protein. **B** The graph shows the DYS protein molecular weight average (kDa, Y-axis) across deletion groups (X-axis). The black bar represents the molecular weight of the full length DYS average in healthy donor cells, grey bars refer to treated patient cells

Golodirsen had already been proven to significantly restore the dystrophin protein on post-treatment muscle biopsies in patients enrolled in the clinical trial NCT02310906 [23]. Here, we used fibroblasts that were MyoD-differentiated into myotubes isolated from the same patients at baseline; the successful myogenic conversion was tested by immunohistochemistry using antibodies against desmin and myosin heavy chain as described in Rossi et al. 2023 [21]. The aim was to confirm the expected size shift of the restored Becker-like protein in patients with different deletions. We also wanted to verify the golodirsen efficiency in increasing dystrophin levels in myotubes, the most common cellular model used in the development of new antisense therapies for DMD [27]. Proteins were extracted from all treated and non-treated patient cultures and measured by capillary western immunoassay (Wes), using two capillary Wes strategies with myosin heavy chain (MF20-Ab) as a normalising protein and protein extract from a pool of healthy controls’ fibroblast MyoD differentiated as a normal control reference. Specifically, MF20 and DYS standard calibration curves (Figure S5 A and B, respectively) were used to assess the restored protein levels in all treated and untreated samples. Most of the treated cultures (# 23) had significant restoration of DYS protein (two tailed *P*-val < 0.0001) with a range of change from non-treated between 0.25 and 2.9% and an average of 1.48%. (Fig. 3A). A single case, patient 101 − 023 exhibited a dystrophin protein restoration of 5.23% that is notably higher and beyond the range detected among the other patients. This value was then omitted from the average calculation due to its high standard deviation. We could not detect DYS production in patient # 101-003 after 5 independently Wes analyses. However, this sample also failed to produce the control MF20 protein in all the 5 independent replicate experiments, suggesting that these results are due to technical problems in achieving myogenic differentiation, rather than a lack of the ability to restore DYS in this patient. The untreated patient cells, our experimental baseline, had no trace of dystrophin proteins with the only exception of the short Dp71 isoform, previously demonstrated to be transcribed in these cell lines [21] (data not shown). Finally, the molecular weight of the restored DYS appears to be consistent with the expected size of the dystrophin protein lacking the exons involved in the deletion and the skipped exon 53 (Fig. 3B). As reported previously [28], due to a ladder underestimation for proteins larger than 280KDa, Wes detects the full-length dystrophin isoform at around 300KDa (HD = 308KDa in our experiments) instead of the expected 427KDa. For this reason, we cannot precisely assess the exact size of the restored protein, but a size-prediction was made using the mathematical proportion of DYS full length amino acid numbers (AA): Wes MW in HD = restored DYS AA: Wes MW in patients.

**Fig. 4:**
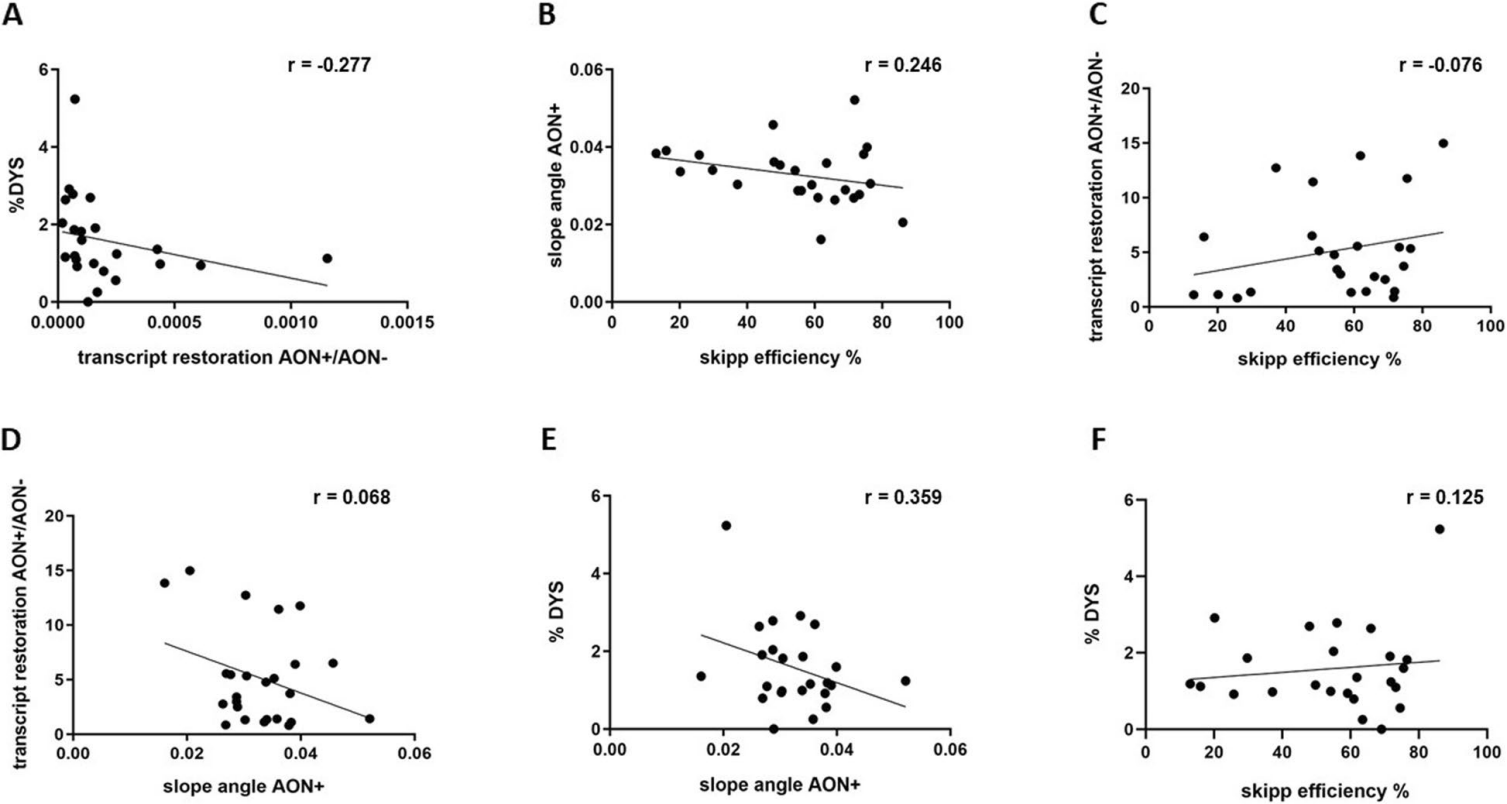
Pearson correlation analyses. All graphs are obtained by GraphPad software version 9; r-values for each Pearson analysis are reported on the upper right side of the relative graph. **A** Correlation between amount of restored transcript (X-axis) and amount of restored dystrophin protein (%DYS) (Y-axis) *p*-val = 0.19. **B** Correlation between skipping efficiency percentage (X-axis) and restored transcript imbalance (slope angle AON+) (Y-axis) *p*-val = 0.235). **C** Correlation between exon skipping efficiency percentage (X-axis) and amount of restored transcript (Y-axis) *p*-val = 0.716. **D** Correlation between restored transcript imbalance (X-axis) and amount of restored transcript (Y-axis) *p*-val = 0.744. **E** Correlation between transcript imbalance restoration (X-axis) and amount of restored dystrophin protein (Y-axis) *p*-val = 0.08. **F** Correlation between skipping efficiency percentage (X-axis) and amount of restored dystrophin protein (Y-axis) *p*-val = 0.559

Interestingly, despite the appreciable exon skipping efficiency found in our treated cellular model, followed by improved transcript imbalance and protein restoration elicited by golodirsen administration in the majority of patients, we did not find any correlation among these biological variables. We tested the correlation between amount of restored transcript (total amount of *DMD* transcript in treated minus non treated samples) versus amount of restored dystrophin protein (Fig. 4A) (*r*= −0.277, *p* = 0.19), skipping efficiency versus transcript imbalance restoration (Fig. 4B)(*r* = 0.246 *p* = 0.235), skipping efficiency versus amount of restored transcript (Fig. 4C)(*r*= −0.0764 *p* = 0.716), amount of restored transcript versus transcript imbalance restoration (Fig. 4D)(*r* = 0.068 *p* = 0.744), transcript imbalance restoration versus amount of restored dystrophin protein (Fig. 4E)(*r* = 0.359 *p* = 0.08), skipping efficiency versus amount of restored dystrophin protein (Fig. 4F)(*r* = 0.125 *p* = 0.559).

### Deletions breakpoints do not show any common cluster but tend to map in intronic genomic regions rich in repetitive DNA elements

**Fig. 5:**
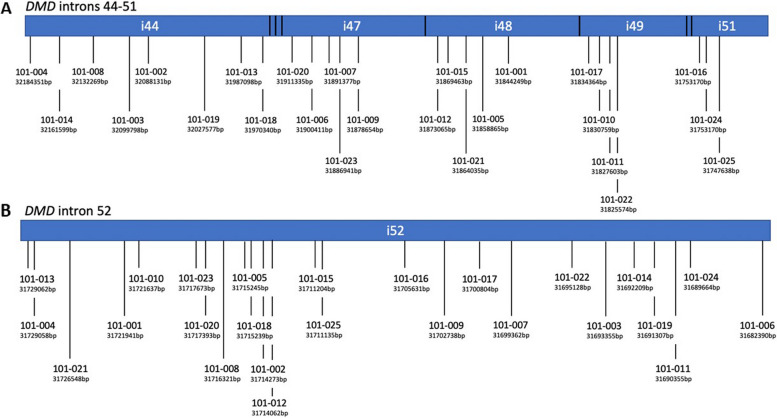
Deletion breakpoints coordinates. **A** Schematic representation of patient upstream breakpoints (vertical lines) among the *DMD* introns (blue bar) 44, 47, 48, 49 and 51 and **B** downstream breakpoints in the *DMD* intron 52. For each breakpoint, patient code and distance (bp) from the upstream (**A**) or downstream (**B**) relative exon are specified. Vertical lines on the figure are non-uniformly distributed along blue bars to represent the relative distance of breakpoints to each other. The length of the blue bars is not proportional to the correspondent intron size, but it is only intended to show all the patients’ breakpoints

In order to assess if different genomic configuration could account for the exon skipping inter-patient variability observed in-vivo [23], we investigated our patients by whole genome sequencing (WGS). Although our cohort is composed of patients carrying only 5 different types of deletion (i.e. D45-52, D48-52, D49-52, D50-52, D52) we found that all breakpoints have unique coordinates either when occurring in the upstream introns (Fig. 5A) or in the common downstream intron 52 (Fig. 5B). The breakpoints distribution between the two-intron extremities, the 5’ and the 3’ end, both for the deletion affecting the upstream and downstream introns, were relatively homogenously distributed. Specifically in our cohort, 10 patients had breakpoints closer to the 5’end and 9 patients to the 3’end of the upstream deletion introns, while in the shared intron 52, 14 patients had breakpoints close to the 5’ end and 8 closer to the 3’ end. The remaining patients had breakpoints occurring in the middle of the introns, more precisely 6 breakpoints in the upstream deletion introns (2 in exon 52, 1 in exon 49, 2 in exon 48, 1 in exon 45) and 3 in the downstream shared intron 52 (Table S5).

Repeated mask analysis identified several transposable and a few repetitive elements either near to, or including, patient breakpoints. Specifically, we found that only 4 patients had breakpoints not surrounded by any elements, whereas the majority (13 patients) had one breakpoint nearby or inside DNA repetitive elements; the remaining 8 patients had both breakpoints involved or close to DNA repetitive elements. Among the identified DNA elements surrounding patient breakpoints (around 150 bp from the breakpoint) we found 16 elements belonging to the long interspersed retrotransposable elements (LINEs) family, 7 elements belonging to the short interspersed nuclear elements (SINEs) family, 7 long terminal repeat (LTRs), 5 DNA transposons which transpose through the “cut-and-paste” mechanism (hAT, Tc1 and PB superfamilies) and 2 simple repeats (Table S5). No further sequence variations were found around breakpoint regions, except for patients 101-005 and 101 − 018 carrying a single base deletion(delG) located respectively, 151 bp and 20 bp from their downstream breakpoint (data not shown).

### RNA in-situ analysis suggests a preferential cytoplasmatic localization of therapeutically skipped transcripts, but with inter-patient variability

In an exploration of *DMD* transcript intra-cellular localization, a study was conducted on a small cohort of these patients (#8, see material and methods) to investigate whether transcripts with re-established frame following AON administration exhibit different localization patterns than out-of-frame transcripts. Recent findings indicate a drastically reduction of *DMD* mRNA in the cytoplasm of DMD patients [29] and animal models (*mdx* mouse and canine DMD model) [17, 19], coupled with a preference nuclear localization. Using BaseScope probes specific for golodirsen-mediated skipped and non-skipped transcripts, we conducted in-situ RNA hybridization on treated myotubes .

Commercial probes targeting PolR2a and DapB served as positive and negative controls, respectively. Signals, represented by red dots were quantified from 16 areas (0.31 × 0.23 mm²) for each patient sample. Counts for both custom probes were categorized based on their sub-cellular localization, distinguishing between cytoplasmic (CYT) and nuclear (NUC), and normalized to the total count in the same area. Our observations align with existing literature, confirming a nuclear preference for non-skipped (out-of-frame) transcripts in DMD patients, contrasting with the predominant cytoplasmic localization observed in healthy controls’ cells. Additionally, consistent with a recent report by Falzarano et al. [29], we observed larger and smaller dots (Figure S6), with the first predominantly in nuclei and the second in the cytoplasm. As a novel observation, we noticed an opposite trend in localization for the skipped transcript (in-frame transcript-therapeutically mediated), which exhibited a preferential cytoplasmic localization. Among the 8 patients tested, 5 displayed a ratio of skipped to non-skipped transcripts greater than 1 in the cytoplasm and less than 1 in the nuclei: specifically, the skipped transcript showed a1.4 to 3.6-fold higher cytoplasmic localization compared to nuclear localisation (Table 1). Conversely, the remaining three patients had skipped transcript preferentially localised in nuclei, with ratios of 1.3, 2.1 and 5-fold higher than in the cytoplasm (Table 1). Intriguingly, these three patients carrying the larger deletion, ex45-ex52, and have demonstrated low protein restoration in post-treatment muscle biopsies during the clinical trial [23]. In our analysis, we considered the sub-cellular trafficking of *DMD* transcript to be altered in those samples showing a ratio greater or equal to 1.5, with lower values suggesting only a modest trafficking impairment (Table 1, column b).

**Table 1:** DMD transcript intra-cellular preferential localization

Patient code	CYT VS NUC^a^	Ratio^b^
101 − 011	1.3 vs. 0.9	1.4 CYT
101 − 020	1.4 vs. 0.7	2 CYT
101 − 017	1.1 vs. 0.5	2.2 CYT
101-006	1.5 vs. 0.4	3.6 CYT
101 − 018	0.9 vs. 1.2	0.8 NUC
101-008	0.9 vs. 1.9	0.5 NUC
101 − 014	0.4 vs. 2	0.2 NUC
101 − 013	2 vs. 0.8	2.5 CYT

^a^column “CYT VS NUC” list the normalized number of BaseScope dots found in cytoplasm (first value) and in nuclei (second value), using respectively the following formula:$$\begin{array}{cc}\frac{\mathrm{Cytoplasmatic}\;\mathrm{dots}\;\mathrm{skipped}}{\mathrm{Total}\;\mathrm{dots}\;\mathrm{skipped}}/&\frac{\mathrm{Cytoplasmatic}\;\mathrm{dots}\;\mathrm{unskipped}}{\mathrm{Total}\;\mathrm{dots}\;\mathrm{unskipped}}=\mathrm{CYT}\\\frac{\mathrm{Nuclear}\;\mathrm{dots}\;\mathrm{skipped}}{\mathrm{Total}\;\mathrm{dots}\;\mathrm{skipped}}&/\frac{\;\;\;\;\;\;\;\;\;\;\;\mathrm{Nuclear}\;\mathrm{dots}\;\mathrm{unskipped}}{\mathrm{Total}\;\mathrm{dots}\;\mathrm{unskipped}}=\mathrm{NUC}\end{array}$$

^b^column “Ratio” lists the ratio between cytoplasmatic and nuclear transcripts and the preferential localization of the more expressed transcript.

## Discussion

In 2019, the Food and Drug Administration (FDA) gave accelerated approval to golodirsen for the treatment of DMD patients with eligible deletions, based on the increases of dystrophin expression observed in the clinical study SRP-4053, in which golodirsen resulted in an unequivocal increase in protein expression confirmed with western blot and semiquantitative immunohistochemistry [14], despite a degree of inter-patient variability [23]. A deeper investigation of the mechanism of action and effects of golodirsen could provide information to elucidate the observed inter-patient variability and to achieve valuable information for developing new AON for the DMD treatment. Variability of dystrophin restoration is not uniquely found after golodirsen, and has indeed been observed after eteplirsen [30] and viltolarsen therapy [31].

In this study, we aimed to better understand the pharmacodynamic actions of golodirsen by comparing treated and non-treated myogenic cells isolated from 25 DMD patients recruited in the original clinical study [23]. Patients’ fibroblasts, collected at the clinical trial baseline, MyoD-transduced and differentiated into myotubes were treated twice with golodirsen in vitro [21] showing higher levels of exon 53 skipping than those reported in post-treatment muscle biopsies from patients in the in-vivo study [23]. Indeed, while in the in-vivo study, the mean percentage of exon skipping increased to 18.95% after treatment (week 48), here we found a mean increment of 54.5% with an upper range value of 86.16% in treated cultures. These results are not unexpected and likely related to the improved delivery, Endoporter mediated, in the cell culture system compared to the in-vivo study and the well-known delivery efficacy discrepancy between in-vitro studies and in-vivo models [22]. Of interest, the exon skipping efficiency observed in our DMD patient cells was not replicated in healthy control cultures (despite the Endoporter contribution) which, in agreement with literature, showed extremely low levels of exon skipping (average exon skipping activity of 0.42%). Previous studies have indeed reported a similar trend both for PMO and 2′-O-methyl-phosphorothioate (2OMePS) antisense chemistries administrated to control human myotubes [24–26]and wild-type mice [26]. On the other hand, a recent AON chemistry, PPMO, showed a different effect on WT mice reaching nearly 100% of exon-skipping after systemic treatments [32]. While the precise mechanism for these different results between DMD and control cells are not known, a possible explanation could be the defect in cell permeability in dystrophic muscle fibres or myotubes that may increase the PMO and 2OMePS delivery efficiency in patients and mdx mice [33]. This mechanism does not appear to be relevant for the PPMO chemistry that has a cell-penetrating peptide conjugated in its backbone [34]. A previously suggested hypothesis involving nonsense-mediated decay (NMD), actively eliminating transcripts with altered frames due to exon skipping in healthy cells, fails to elucidate the observed differential behaviour among the analysed AON chemistries. Indeed, if the NMD was a highly efficient phenomenon, we would observe low or null exon skipping efficiency in healthy cultures regardless of the chemistries of the AON used, with no differences between PMO and PPMO.

Thanks to the unique FluiDMD cards’ design, we were able to confirm the precise action of golodirsen in selectively inducing skipping of exon 53 only and exclude the presence of other unspecific skipping products; this is the first time such a comprehensive analysis of the in clinical-used-ASO induced splicing has been performed. Likewise, the restored protein sizes were evaluated by Wes and they were proportional to the product of the *DMD* gene based on the genomic deletions of the patients studied.

In this study we demonstrated that golodirsen-treated cultures consistently and significantly express more *DMD* Dp427 transcript than non-treated patient cultures, even if, as expected, the transcript expression was not homogeneous throughout the *DMD* length, due to the *DMD* transcript imbalance [16]. The limited understanding of the molecular mechanisms behind the transcript imbalance phenomenon, even if originally described in 1995 [16], may need to be considered more broadly in the field of AON therapy. Neglecting the presence of *DMD* transcript imbalance could result in erroneous prediction of the level of protein restoration of AON’s therapies in the human, irrespective of the backbone of the AON, and a misestimation of the skipping efficiency and transcript expression. Here, for the first time in human cells we demonstrated that an AON is able to significantly (< 0.0001) restore the transcript imbalance, decreasing its rate in treated versus non-treated DMD cultures, even if did not completely recover the transcript imbalance in patients’ cells to healthy control levels. Spitali and colleague [18] previously investigated the effect of a PMO AON in *mdx* mice, finding no restorative effect of the antisense on the transcript imbalance rate in-vivo. The discrepancy between our finding and those of Spitali at al. might be attributable to the different transcript imbalance rate caused by the dissimilar type and localization of mutations between the *mdx* mouse model (nonsense in the *Dmd* exon 23) and our patient cohort (deletions between *DMD* exon 45–52). Additionally, the divergence in results might be due to the different biological context and complexity of the models used: their in-vivo model versus our cellular model. Conversely, our findings align to some extend with what previously reported by Anthony et al. [35], where BMD patients carrying in-frame mutations and having a milder phenotype showed a reduced transcript imbalance level compared to the related out-of-frame deletion patients. Indeed, the therapeutical frame restoration leads to the increment of the *DMD* transcript stability, mirroring the relative stability observed in BMD patients. However, in this relatively limited cohort of DMD patients, we found no clear correlation between levels of exon skipping efficiency and transcript imbalance restoration. Similarly, we did not found correlation even between dystrophin protein restoration and exon skipping efficiency or the imbalance rate. Probably, a simple pairwise relationship, evaluated in a correlation analysis, cannot explain the complexity and cover all factors involved in the multiple biological processes relevant for the efficiency of protein restoration. In addition, protein stability and turnover are likely to vary between an in-vivo differentiated cellular model and mature muscles.

Our work also identifies other important aspects in addition to delivery of AONs to skeletal muscles when treating DMD patients [36]. An issue that might influence the efficiency of exon skipping therapies was recently highlighted in animal model and in-vitro human studies [17, 19, 29]. Both DMD models showed an unbalanced *DMD* transcript localization between nuclei and cytoplasm with a predominant nuclear compartmentalization, suggesting that the characteristics of *DMD* transcript, specifically the mutated *DMD* pre-mRNA, affects its own export efficiency into cytoplasm. Our results of in-situ RNA hybridization experiments confirmed these observations and seems to align with that intriguing speculation. In fact, we observed a different intra-cellular localization between therapeutically-in-frame and mutant out-of-frame transcripts, with 6 of the 8 analysed patients showing a trafficking towards the cytoplasm associated with the therapeutically-in-frame transcripts. The limited number of patients analysed and the technical limitations posed by BaseScope experiment performed on primary differentiated human myotubes should be considered as exploratory in nature. They nevertheless highlight the importance of pursuing further studies, in a therapeutically corrected reading frame context, to better understand the variability observed following AON treatments. Indeed, despite several hypotheses, there has never been a study in which the difference in patient responses to AON treatment was completely addressed [37]. Inter-patient variability was also confirmed in the clinical study SRP-4053 [23] and in the vitolarsen paper [31] in the protein restoration levels among the enrolled subjects. Although the majority of patients showing in vivo lower protein restoration carried an ex45-ex52 deletion, this association between patient genotype and lower restored protein level was not confirmed by this in-vitro study. In contrast, Takizawa et colleagues reported that the exon 51 skipping efficiency varied according to the *DMD* mutation, with higher skipping efficiency associated to exon 49–50 deletion [38] in their in-vitro study, although only a small number of subjects were studied (#7).

Elegant work from Gazzoli et al. [39] speculates about an exon block removal mechanism occurring in the *DMD* gene, where the exons included between exon 45 to exon 49 are spliced together within the same block and exon 44 instead, is joined to exon 45 by an intermediate-speed non-sequential splicing process. Moreover, intron 44 is apparently removed by several (#59) nested and recursive splicing steps [39]. These observations may suggest that deletions involving intron 44 are associated with a less efficient therapeutic restoration [40]. On the other hand, all of our patients conserved the unique pausing site in intron 52, previously identified by Gerardi et al. [41] having their breakpoints on intron 52 located upstream the pausing site, avoiding any interference with the dystrophin transcriptional dynamics regulated by this region [29].

From a genomic point of view, our cohort represents the typical DMD cohort, having deletions occurring in the common hotspot, between exon 45 and exon 52 [42], with breakpoints randomly distributed along the involved introns instead of being clustered at a single site, as previously observed [43, 44]. Interestingly we noticed an overall equal distribution of breakpoints between the two extremities of introns, with some breakpoints occurring in the middle of the introns. This is in contrast to what has been reported for intron 44, that shows a preferential breakpoints localization at 50kb [45] or 100 kb of its distal (3’) region [46]. This same intron 44 distal region has the highest ratio of repetitive elements of the entire *DMD* gene [46–49], supporting the idea of their direct involvement in the *DMD* gene rearrangements by facilitating and mediating the non-homologous end joining (NHEJ) mechanism [42, 43]. Indeed, while the average frequency of repetitive elements in the entire *DMD* gene is approximately 35.6%, most breakpoints map to one or more of them [43]. This is in agreement with our cohort, where the majority of the patients (84%) have at least one breakpoint in the proximity of one, two or three repetitive elements. On an overview of the breakpoints, we found no association between patient treatment response and deletion size or breakpoints localization relative to neighbouring exons.

## Conclusion

In this study we demonstrate the high efficacy of golodirsen in exon skipping and transcript expression in-vitro and confirm its specificity in targeting and removing only exon 53 from the *DMD* transcript. Indeed, no aberrant-spliced transcripts were found in the treated cultures and, consequently the restored proteins were of the expected molecular weight. Similarly to what was found in our previous in-vivo clinical study, we noticed a substantial difference between the exon skipping efficiency and the level of dystrophin restoration even if, in the current study, the exon-skipping efficiency was greater than the one of the in-vivo study due to the local delivery of the PMOs. For the first time in an in-vitro human primary cell model, we demonstrated the ability of an AON to significantly restore the transcript imbalance in treated versus non treated DMD patient cells. This finding reveals that restoring the correct reading frame promotes the *DMD* transcript maturation and consequently the substrate for protein production. Likewise to the in-vivo study, we also observed inter-patient variability and modest protein restoration levels despite here the efficiency of exon 53 skipping was significantly higher in our in-vitro study. On the other hand, the variability observed does not appear to correlate with any patient characteristic, including their intronic breakpoints which we fully characterised. Observing an inter-patient variability in an in-vitro study, where the delivery issue has little impact, underlines the crucial role of the biological mechanisms upstream of translation of the DMD protein. These mechanisms should be better investigated to overcome some of the bottlenecks identified in antisense clinical trials, in order to improve the therapeutic outcomes of both the current and novel AONs entering the clinical arena.

Limitations of the study: The in-situ RNA hybridization technique used in this project, BaseScope^™^, was not designed for use in primary cell cultures, as specified by the technique’s developer (ACD | Bio-Techne). Consequently, we encountered technical challenges that combined with the low number of patients investigated, did not allow for further interpretation of the experiment outcomes. Additional analyses are required to better investigate the biological mechanisms upstream of protein production that limit the efficiency of AON-mediated protein restoration.

## Materials and methods

### Cells

**Fig. 6:**
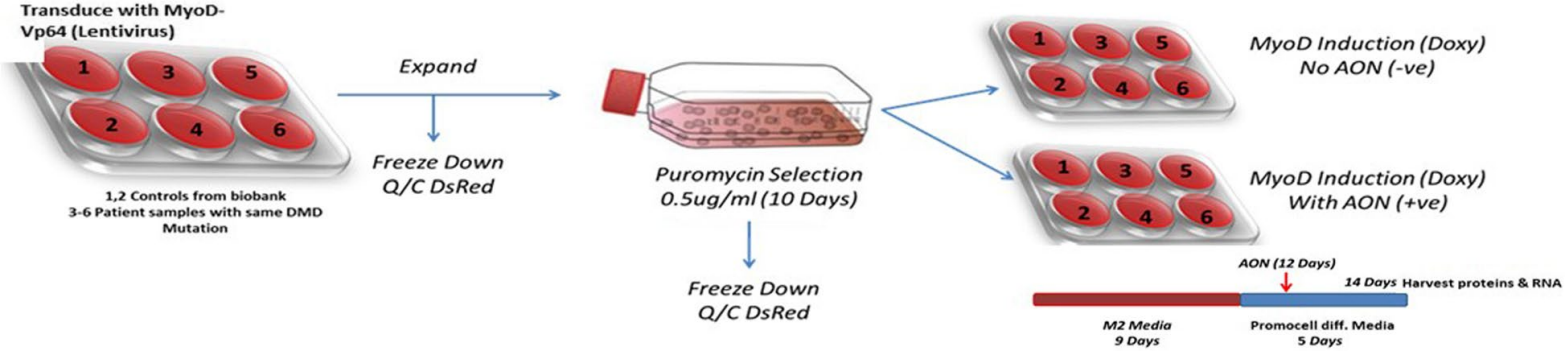
Scheme of the cellular experiments performed: including cells culturing, differentiation, and treatment

Fibroblasts from 25 DMD patients enrolled in the clinical study SRP-4053 (registered at clinicaltrials.gov as NCT02310906) and from 3 paediatric (5, 7, and 9 years old) non-neuromuscular disease donors (ligamentous laxity, motor delay, spastic diplegia) were isolated from skin biopsies collected after obtaining written informed consent and, for the DMD subjects, at the baseline of the clinical study. This work was performed under the NHS National Research Ethics: setting up of a rare diseases biological samples bank (biobank) for research to facilitate pharmacological, gene and cell therapy trials in neuromuscular disorders (REC reference number: 06/Q0406/33), and the use of cells as a model system to study pathogenesis and therapeutic strategies for Neuromuscular Disorders (REC reference 13/LO/1826). All fibroblasts were MyoD-differentiated into myotubes by lentiviral vector following the protocol described in Rossi et al. 2023 [21]. Cells were expanded and differentiated in two 6-well-plates, one treated with golodirsen (+ AON) and one non-treated and used as control (-AON). Golodirsen was administrated twice to + AON cultures, at day 6 and at day 12 of the trans-differentiation process with 24 h and 48 h of incubation, respectively. Specifically, 10ul of golodirsen was administrated on each 6-well in combination with 6ul of Endoporter (PEG, Gene-Tools). RNA and proteins were collected at day 14 of the trans- differentiation process, and at day 8 from the first and at day 2 from the second administration of golodirsen, from treated and non-treated cultures. Three wells for each plate were used to isolate RNA and the other three for protein. 2 controls were cultured twice to have a total of 5 healthy samples: 3 biological and 2 technical replicates, all treated in parallel with DMD patients (Fig. 6). Detailed protocols of golodirsen administration, tests of the successful myogenic conversion of fibroblasts to myotubes, RNA and protein isolation and treatment are described in Rossi et al. 2023 [21].

Data of dystrophin increment over baseline from our previous in-vivo study [23] were used to subgroup patients into three categories of responders: good, intermediate, and low. Specifically, we defined “low responder” patients as having no restoration of dystrophin over baseline, “intermediate responder” patients with a level of restoration of less than 1%, and “good responders” patients with restored dystrophin level of 1% or more.

### RNA analyses

Total cDNAs were initially tested by PCR following manufacturer’s instructions (cat. No. 10342053, Invitrogen, Waltham, MA, US) and the amplified products visualized on a 2% agarose gel. Custom primers designed to overlap both patients deleted exon/s and exon 53 were used; the revers primer was unique for each patient and located on exon 54 (primer sequences available on request). Exon-skipping efficiency and transcript amount were quantified by qPCR. Specifically, 2 inventoried TaqMan systems (Thermo Fischer Scientific, cat. no.4331348) complementary to the exon-exon junction 53–54 (Hs01049452_m1) and 55–56 (Hs01049450_m1) of human DMD Dp427 and 1 commercial TaqMan system for the human ACTB gene (Hs01101944_s1), were used on StepOnePlus™ Real-Time PCR machine (Applied Biosystems). Raw amplified data were loaded into LinRegPCR software (version 2014.x) to calculate starting concentration per sample (N0) corrected by Taqman system PCR efficiency [50, 51]. In order to perform the most accurate analysis as possible, we did not consider the quantification cycles (Ct or Cq), that are strongly dependant on amplification efficiency, but the starting concentration per sample or target quantity (N0) for the DMD full length transcript (referring to TaqMan system exon 55–56), the DMD skipped transcript (referring to TaqMan system exon 53–54) and the housekeeping gene (ACTB). Specifically: N0 values obtained from the TaqMan exon 53–54 and exon 55–56 systems were divided for the housekeeping gene ACTB N0 values to assess the DMD non-skipped (N0 _ex53_) and the total (N0 _tot_) transcript amount, respectively, for each patient. Exon 53 skipping efficiency percentage was calculated using the formula: 100-[(N0_ex53_/N0_tot_)_+AON_ / (N0_ex53_/N0_tot_)_-AON_ *100]. The exon-exon junction 55–56 was selected as reference to the total transcript because of its genomic proximity to exon-exon junction 53–54, to avoid the transcript imbalance interference. Transcript amount for each samples was assessed on treated cells (+ AON) and on non-treated cells (-AON) by the formula: (N0_tot_)_+AON_ / (N0_tot_)_-AON_. On the graph in Fig. 1B, the transcript expression in non-treated cells (N0_tot_)_-AON_ was set to 1 to better appreciate the transcript increment after AON treatment. PCRs and qPCRs were performed separately for all technical replicates and the visualized values are the average of the three replicates for each sample.

Custom FluiDMD cards were prepared and run on QuantStudio™ 7 Flex Real-Time PCR (Applied Biosystems), and the transcript imbalance calculated as described by Bovolenta et al. 2012 [52]. Briefly, 2.2ul of each replicate were pooled together and 150ng of cDNA mixed with 100ul of Universal PCR Master Mix 2X (Thermo Fischer scientific, cat. no.4304437) were loaded on each Fluidic card port; two consecutive ports were loaded with the same sample to ensure the total DMD transcript coverage. Then threshold cycle (Ct) values obtained after FluiDMD cards’ analysis were plotted in a spreadsheet (Y-axis) along with the corresponding DMD exon-exon junctions (X-axis), for each sample - treated and non-treated. Regions affected by patient mutations and DMD exon 53 were not included in the calculation. The transcript imbalance rate corresponds to the slope angle value of regression lines drawn among Ct. Visual representation of transcript amount decrement through transcript length was obtained by calculating the transcript expression by the 2^^−DDCt^ method described by Bovolenta et al. 2012 [52].

### Wes analysis

For each treated and non-treated sample, total proteins were extracted from each technical replicate (*n* = 3) and pooled together. 4ug of each pool was loaded on Wes system (ProteinSimple, Bio-Techne, USA) following the manufacturer’s instructions and using rabbit anti-dystrophin (ab15777, Abcam, UK, 1/50; ab154168, Abcam, UK, 1:1000), anti MF20 (Developmental Studies Hybridoma Bank, USA, 1:200), anti-rabbit (DM-001), and anti-mouse (DM-002) primary and secondary antibodies. A pool of total proteins extracted from the healthy control non-treated cells were used to build two calibration curves: a 5-point curve ranging from 0.25 to 2 ug and a 6-point curve ranging from 0.025 to 1ug and respectively for MF20 and DYS. The curves were included in all the Wes analysis, to control for muscle content and as reference to allow the calculation using the two capillaries strategy and the formula described by Beekman et al. 2018 [28]. Specifically, to calculate the percentage of restored dystrophin change from baseline in treated patient cells, the following formula was used:

[(%of ctrl DYS/ %of ctrl MF-20) _+AON_ - (%of ctrl DYS/ %of ctrl MF-20) _−AON_] *100.

In the formula the minuend is the restored DYS in treated samples (+ AON) expressed as the normalised percentage of healthy controls (ctrl) and the subtrahend is the DYS in non-treated samples (-AON) expressed as the normalised % of healthy controls. In our experiments the subtrahends were always 0. In patient # 101-002, the lack of signal for both DYS and MF20 proteins, in all 5 independent replicates, was probably due to sample’s degradation.

### WGS

DNA was extracted from patient primary fibroblasts (non-treated and non-differentiated) using the DNeasy Blood & Tissue Kit (QIAgen, cat n. 69504) following the manufacturer’s instructions. DNA fragmentation, WGS DNBs library preparation and sequencing on the BGISEQ-500 platform were performed by BGI (BGI Genomics, MGI) likewise a cleaning data and standard bioinformatic analysis. Bioinformatic analysis included variant calling and annotation (e.g., SNP, insertion or deletion [indel], and CNV) and local realignment around InDels. Coverage, reads quality and, breakpoint coordinates annotation were assessed using Integrative Genomics Viewer (IGV) and the human reference genome assembly GRCh38. Interspersed repeats and low complexity DNA sequences were investigated in sequences located 150 bp downstream and upstream patient deletion breakpoints by RepeatMasker Open-4.0 program [53] using its default parameters. The repetitive elements were annotated categorized by their family.

### BaseScope™

MyoD-transfected fibroblast cells from 8 patients and 1 healthy control cells were seeded and differentiated on 4 chamber Nunc™ Lab-Tek™ Chamber Slide with Permanox Plastic support (Thermo scientific cat. n. 177437PK). For each patient cell culture, golodirsen was administered twice, one at day 5 and one at day 8 of trans-differentiation process and incubated 24 h and 48 h respectively. After 10 days of trans-differentiation, myotubes were fixed, denatured and protease treated using RNAscope H202 and protease reagents (ACD | Bio-Techne, cat n. 322381). Subsequentially, the BaseScope experiment was conducted on processed slides using custom probes and BaseScope Detection Reagents V2-RED (ACD | Bio-Techne, cat n. 323910) following manufacturer’s instructions. Specifically, one of the treated patient chamber myotubes was hybridized with custom probes specific for DMD exon-exon junction 53–54 (non-skipped transcript) and myotubes on the other treated chamber with a probe overlapping patient’s specific deletion plus ex 53, i.e. exon-exon junctions 44–54, 47–54, 49–54 (skipped transcript). The remaining two chambers for each patient were hybridized with positive (PolR2a) and negative (DapB) commercial control probes. Chromogenic signals were detected by Leica DM6B microscope and red dots counted from 16 areas of 0.31 × 0.23mm^2^ for each patient sample. Dots of both custom probes were counted and divided by their sub-cellular localization (cytoplasmatic or nuclear). Cytoplasmatic (CYT) and nuclear (NUC) dot numbers were normalized to the total number of dots counted in the same area, for skipped transcript probe (−53) and non-skipped transcript probe (+ 53). Ratio between skipped and non-skipped transcript was assessed in both subcellular localizations: [CYT _(−53)_/CYT _(+53)_]/ [NUC _(−53)_/NUC _(+53)_]. Dots localized in the perinuclear region were counted as nuclear dots. For all patients and the healthy control, the positive commercial control Pol2R probe identified widespread sparse signals, that were visualized as dots in all cells of the samples tested, while the negative DapB probes returned no dots at all, as expected.

### Statistical analysis

Graphs, Wilcoxon test and Pearson correlation were performed using GraphPad Prism version 9.0.0 (for Windows, GraphPad Software, San Diego, CA, USA, www.graphpad.com). Two tailed p value ≤ 0.05 was considered significant. In the statistical analyses, the slope’s angles calculated from + AON cultures were considered as the transcript imbalance variable, the normalised % of DYS as the restored protein level and levels of DMD mRNA calculated on + AON cultures as the transcript amount. Patient # 101-002, due to technical problems on Wes, was removed from all calculations involving % of restored DYS.

## Supplementary Information

Supplementary Material 1

## Data Availability

All the relevant data and experimental procedures are shown in the article; raw and characterization data can be found in the supplemental information. Also, the datasets and additional raw data used in the current study are available from the corresponding or last author on reasonable request.
