# Supplementary material for: Golodirsen restores DMD transcript imbalance in Duchenne Muscular Dystrophy patient muscle cells
Source: Skelet Muscle. 2024 Nov 29;14:28. doi: 10.1186/s13395-024-00360-4 (PMC11606086; doi:10.1186/s13395-024-00360-4)
Supplement: Supplementary file 1 — Supplementary Material 1 [file 13395_2024_360_MOESM1_ESM.pptx]

## Slide 1
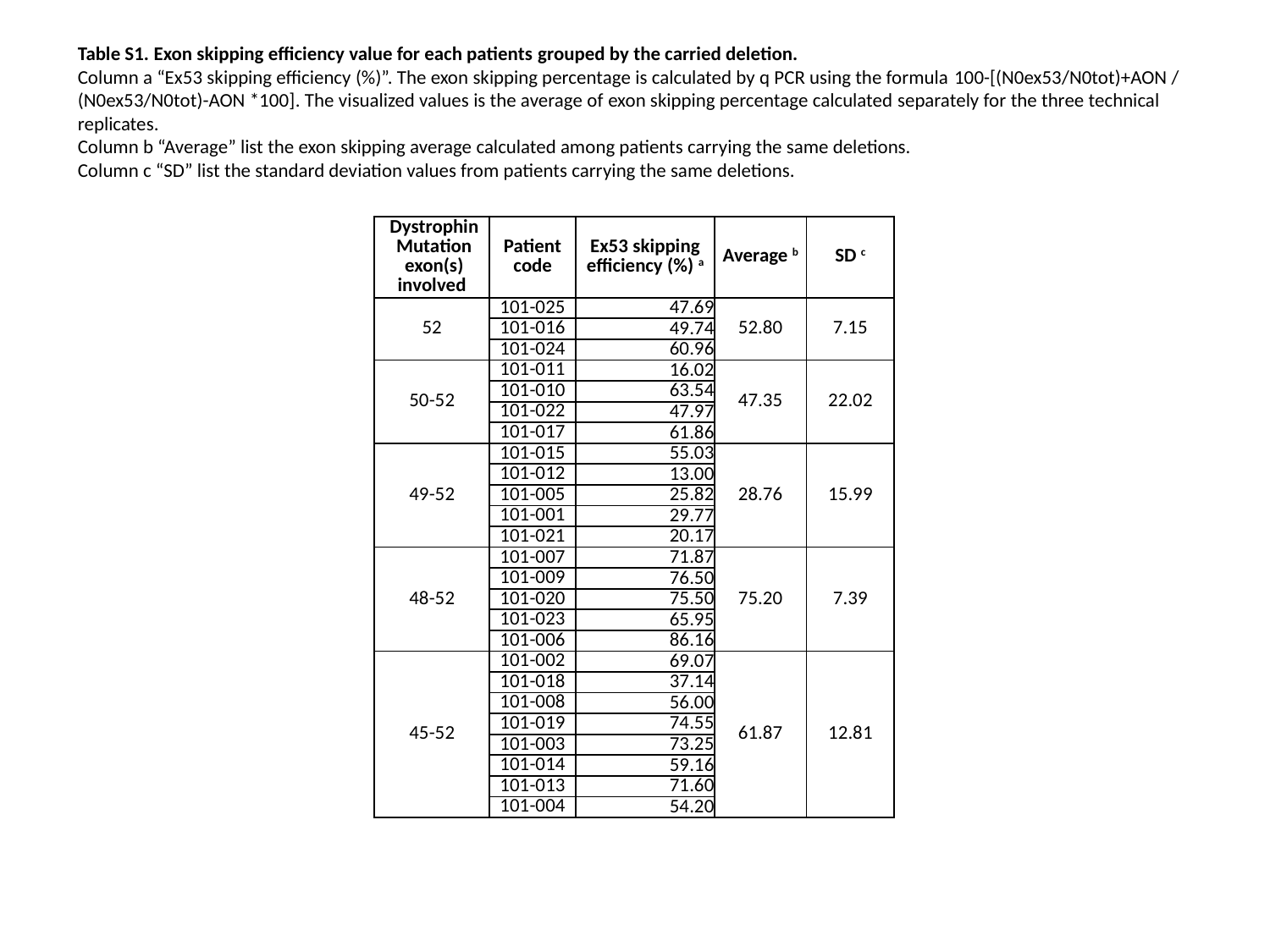

Table S1. Exon skipping efficiency value for each patients grouped by the carried deletion.
Column a “Ex53 skipping efficiency (%)”. The exon skipping percentage is calculated by q PCR using the formula 100-[(N0ex53/N0tot)+AON / (N0ex53/N0tot)-AON *100]. The visualized values is the average of exon skipping percentage calculated separately for the three technical replicates.
Column b “Average” list the exon skipping average calculated among patients carrying the same deletions.
Column c “SD” list the standard deviation values from patients carrying the same deletions.
| Dystrophin Mutation exon(s) involved | Patient code | Ex53 skipping efficiency (%) a | Average b | SD c |
| --- | --- | --- | --- | --- |
| 52 | 101-025 | 47.69 | 52.80 | 7.15 |
| | 101-016 | 49.74 | | |
| | 101-024 | 60.96 | | |
| 50-52 | 101-011 | 16.02 | 47.35 | 22.02 |
| | 101-010 | 63.54 | | |
| | 101-022 | 47.97 | | |
| | 101-017 | 61.86 | | |
| 49-52 | 101-015 | 55.03 | 28.76 | 15.99 |
| | 101-012 | 13.00 | | |
| | 101-005 | 25.82 | | |
| | 101-001 | 29.77 | | |
| | 101-021 | 20.17 | | |
| 48-52 | 101-007 | 71.87 | 75.20 | 7.39 |
| | 101-009 | 76.50 | | |
| | 101-020 | 75.50 | | |
| | 101-023 | 65.95 | | |
| | 101-006 | 86.16 | | |
| 45-52 | 101-002 | 69.07 | 61.87 | 12.81 |
| | 101-018 | 37.14 | | |
| | 101-008 | 56.00 | | |
| | 101-019 | 74.55 | | |
| | 101-003 | 73.25 | | |
| | 101-014 | 59.16 | | |
| | 101-013 | 71.60 | | |
| | 101-004 | 54.20 | | |

## Slide 2
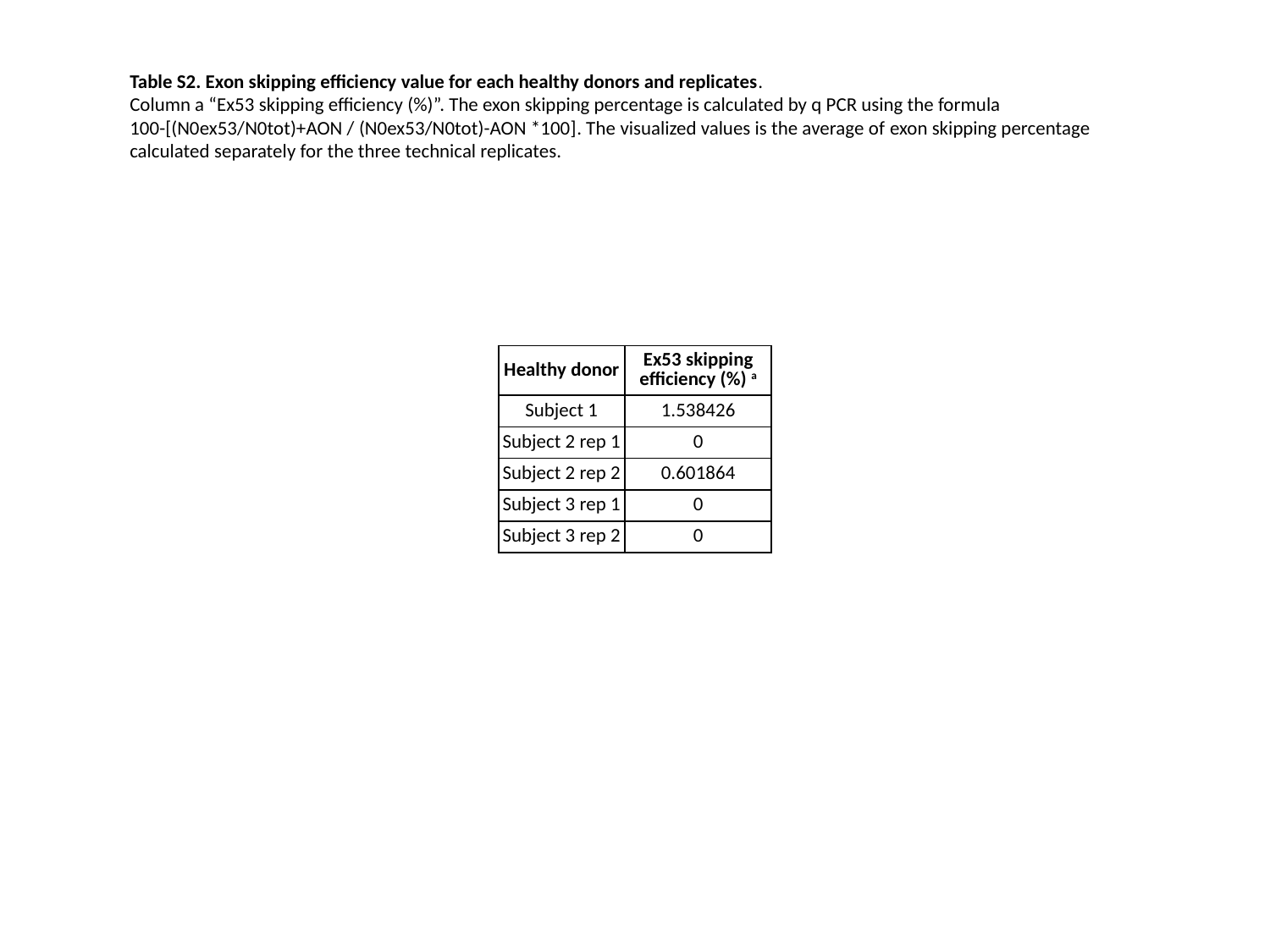

Table S2. Exon skipping efficiency value for each healthy donors and replicates.
Column a “Ex53 skipping efficiency (%)”. The exon skipping percentage is calculated by q PCR using the formula 100-[(N0ex53/N0tot)+AON / (N0ex53/N0tot)-AON *100]. The visualized values is the average of exon skipping percentage calculated separately for the three technical replicates.
| Healthy donor | Ex53 skipping efficiency (%) a |
| --- | --- |
| Subject 1 | 1.538426 |
| Subject 2 rep 1 | 0 |
| Subject 2 rep 2 | 0.601864 |
| Subject 3 rep 1 | 0 |
| Subject 3 rep 2 | 0 |

## Slide 3
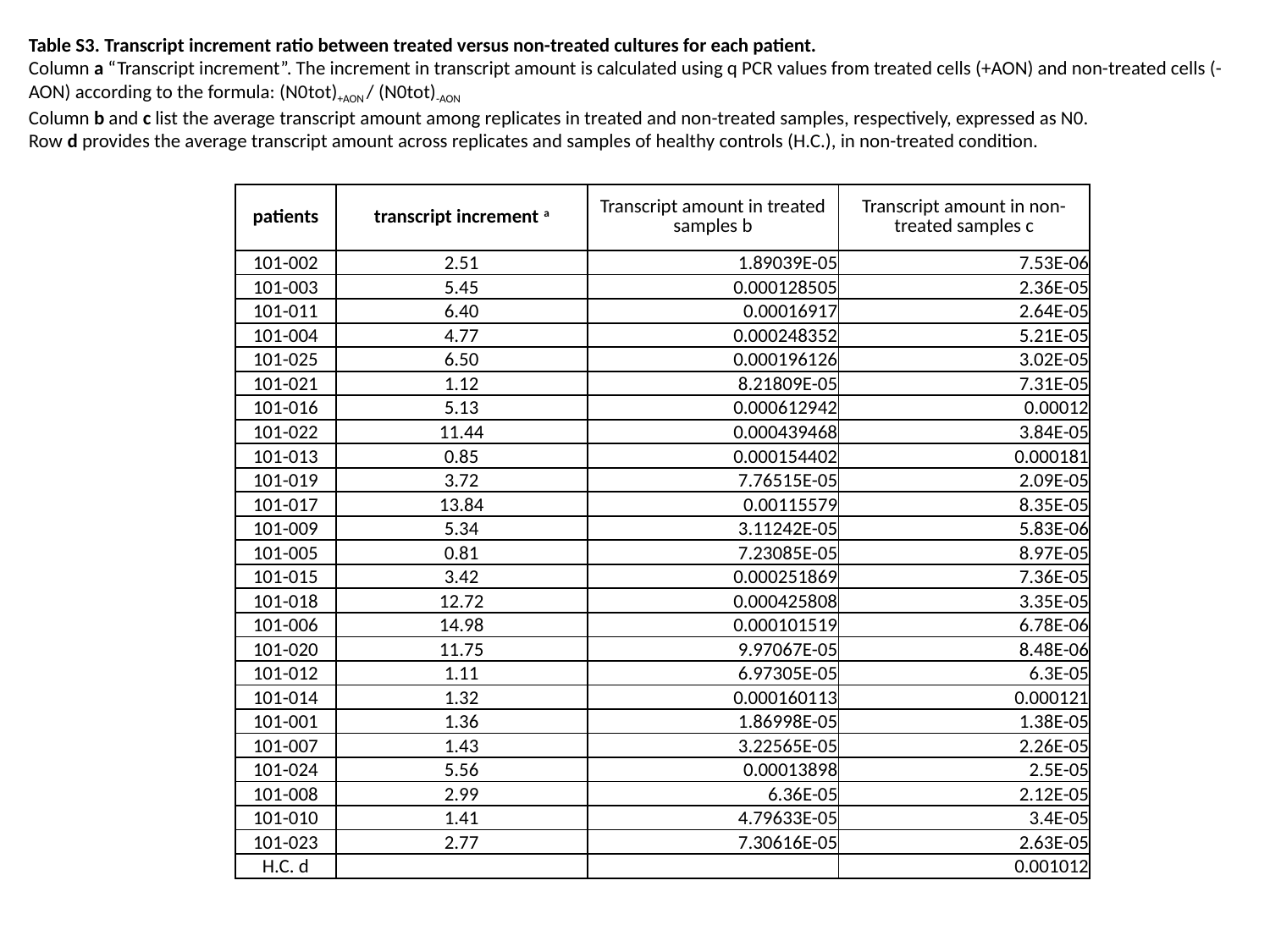

Table S3. Transcript increment ratio between treated versus non-treated cultures for each patient.
Column a “Transcript increment”. The increment in transcript amount is calculated using q PCR values from treated cells (+AON) and non-treated cells (-AON) according to the formula: (N0tot)+AON / (N0tot)-AON
Column b and c list the average transcript amount among replicates in treated and non-treated samples, respectively, expressed as N0.
Row d provides the average transcript amount across replicates and samples of healthy controls (H.C.), in non-treated condition.
| patients | transcript increment a | Transcript amount in treated samples b | Transcript amount in non-treated samples c |
| --- | --- | --- | --- |
| 101-002 | 2.51 | 1.89039E-05 | 7.53E-06 |
| 101-003 | 5.45 | 0.000128505 | 2.36E-05 |
| 101-011 | 6.40 | 0.00016917 | 2.64E-05 |
| 101-004 | 4.77 | 0.000248352 | 5.21E-05 |
| 101-025 | 6.50 | 0.000196126 | 3.02E-05 |
| 101-021 | 1.12 | 8.21809E-05 | 7.31E-05 |
| 101-016 | 5.13 | 0.000612942 | 0.00012 |
| 101-022 | 11.44 | 0.000439468 | 3.84E-05 |
| 101-013 | 0.85 | 0.000154402 | 0.000181 |
| 101-019 | 3.72 | 7.76515E-05 | 2.09E-05 |
| 101-017 | 13.84 | 0.00115579 | 8.35E-05 |
| 101-009 | 5.34 | 3.11242E-05 | 5.83E-06 |
| 101-005 | 0.81 | 7.23085E-05 | 8.97E-05 |
| 101-015 | 3.42 | 0.000251869 | 7.36E-05 |
| 101-018 | 12.72 | 0.000425808 | 3.35E-05 |
| 101-006 | 14.98 | 0.000101519 | 6.78E-06 |
| 101-020 | 11.75 | 9.97067E-05 | 8.48E-06 |
| 101-012 | 1.11 | 6.97305E-05 | 6.3E-05 |
| 101-014 | 1.32 | 0.000160113 | 0.000121 |
| 101-001 | 1.36 | 1.86998E-05 | 1.38E-05 |
| 101-007 | 1.43 | 3.22565E-05 | 2.26E-05 |
| 101-024 | 5.56 | 0.00013898 | 2.5E-05 |
| 101-008 | 2.99 | 6.36E-05 | 2.12E-05 |
| 101-010 | 1.41 | 4.79633E-05 | 3.4E-05 |
| 101-023 | 2.77 | 7.30616E-05 | 2.63E-05 |
| H.C. d | | | 0.001012 |

## Slide 4
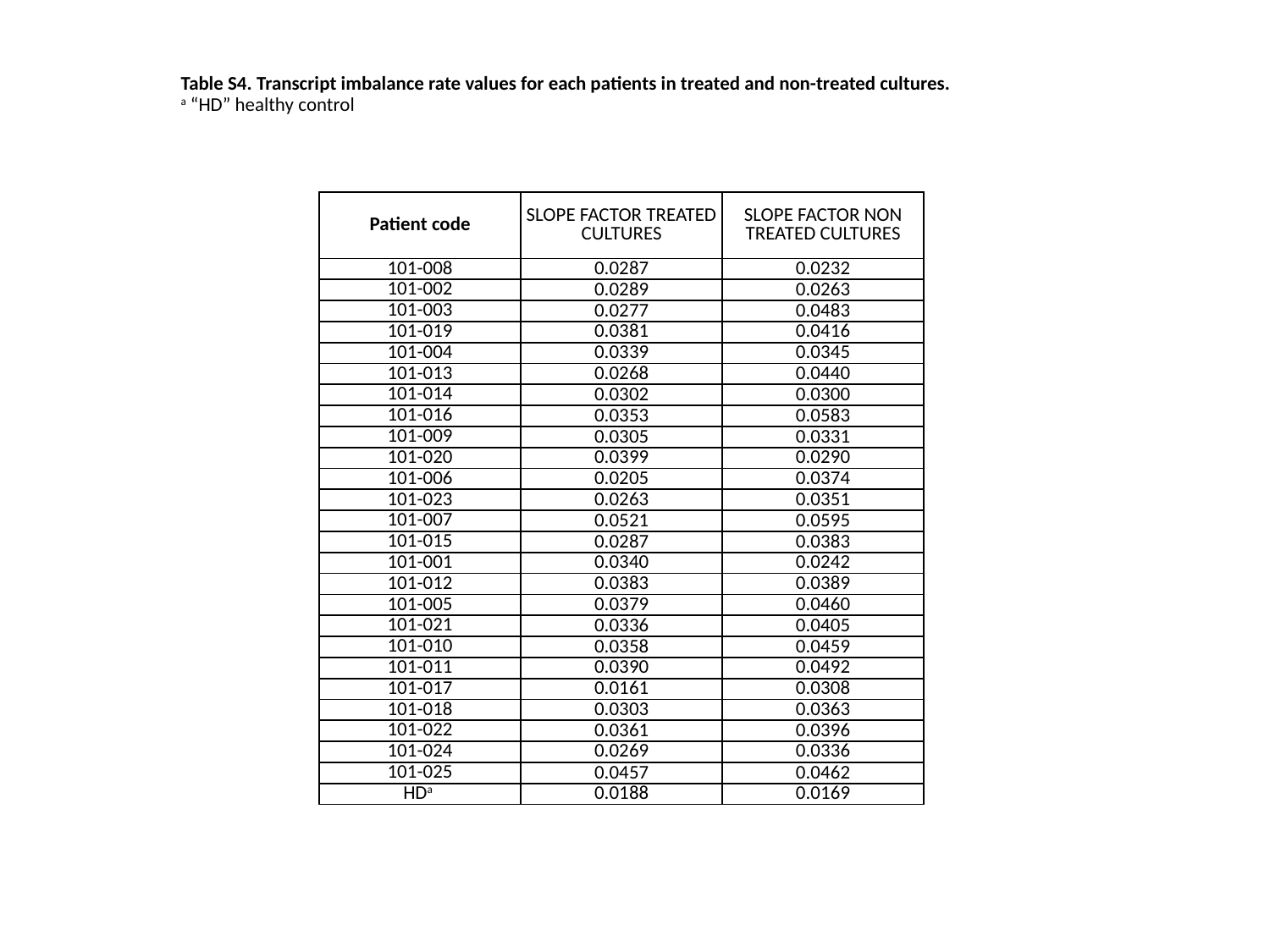

# Table S4. Transcript imbalance rate values for each patients in treated and non-treated cultures. a “HD” healthy control
| Patient code | SLOPE FACTOR TREATED CULTURES | SLOPE FACTOR NON TREATED CULTURES |
| --- | --- | --- |
| 101-008 | 0.0287 | 0.0232 |
| 101-002 | 0.0289 | 0.0263 |
| 101-003 | 0.0277 | 0.0483 |
| 101-019 | 0.0381 | 0.0416 |
| 101-004 | 0.0339 | 0.0345 |
| 101-013 | 0.0268 | 0.0440 |
| 101-014 | 0.0302 | 0.0300 |
| 101-016 | 0.0353 | 0.0583 |
| 101-009 | 0.0305 | 0.0331 |
| 101-020 | 0.0399 | 0.0290 |
| 101-006 | 0.0205 | 0.0374 |
| 101-023 | 0.0263 | 0.0351 |
| 101-007 | 0.0521 | 0.0595 |
| 101-015 | 0.0287 | 0.0383 |
| 101-001 | 0.0340 | 0.0242 |
| 101-012 | 0.0383 | 0.0389 |
| 101-005 | 0.0379 | 0.0460 |
| 101-021 | 0.0336 | 0.0405 |
| 101-010 | 0.0358 | 0.0459 |
| 101-011 | 0.0390 | 0.0492 |
| 101-017 | 0.0161 | 0.0308 |
| 101-018 | 0.0303 | 0.0363 |
| 101-022 | 0.0361 | 0.0396 |
| 101-024 | 0.0269 | 0.0336 |
| 101-025 | 0.0457 | 0.0462 |
| HDa | 0.0188 | 0.0169 |

## Slide 5
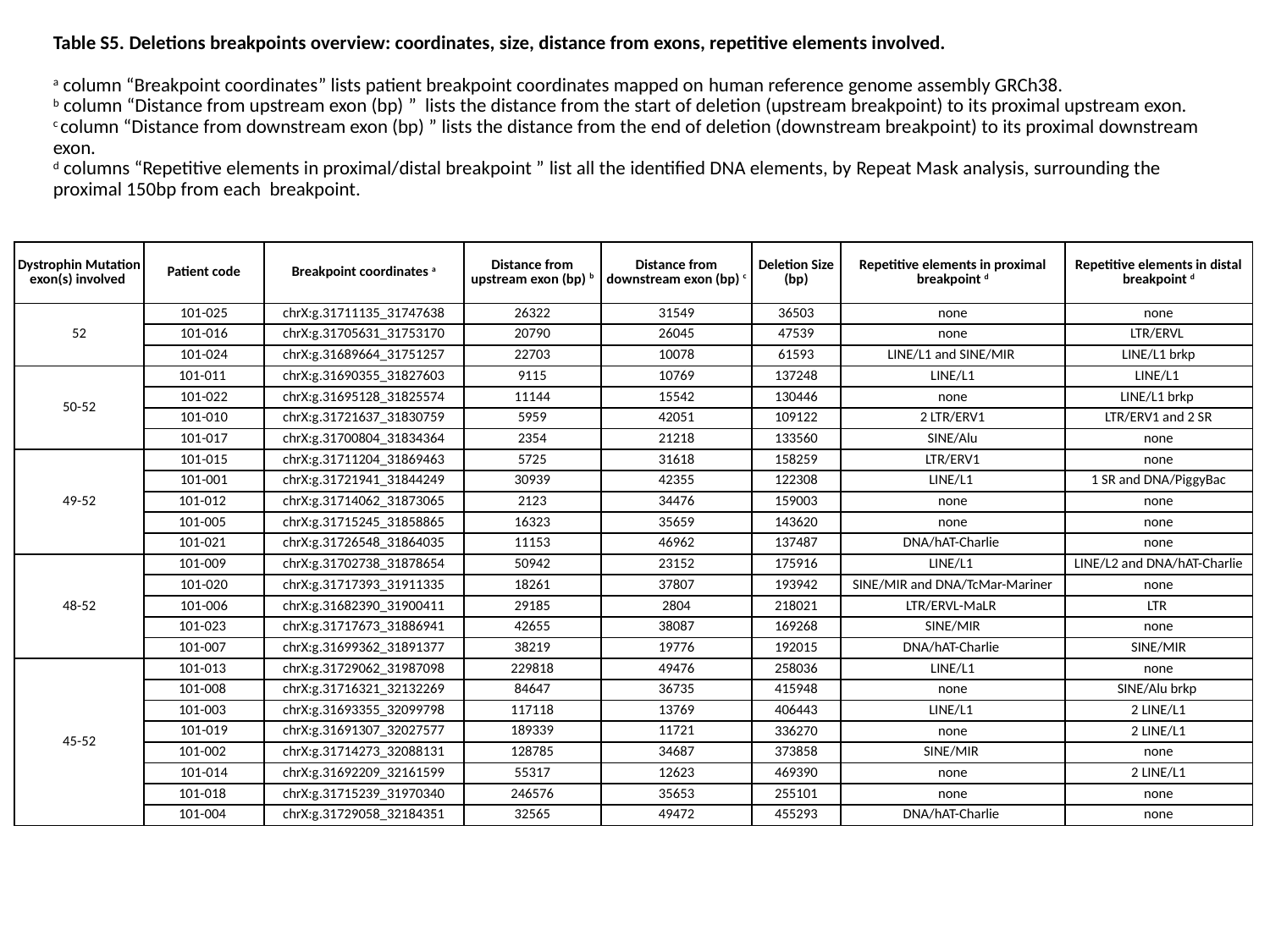

# Table S5. Deletions breakpoints overview: coordinates, size, distance from exons, repetitive elements involved. a column “Breakpoint coordinates” lists patient breakpoint coordinates mapped on human reference genome assembly GRCh38. b column “Distance from upstream exon (bp) ” lists the distance from the start of deletion (upstream breakpoint) to its proximal upstream exon. c column “Distance from downstream exon (bp) ” lists the distance from the end of deletion (downstream breakpoint) to its proximal downstream exon. d columns “Repetitive elements in proximal/distal breakpoint ” list all the identified DNA elements, by Repeat Mask analysis, surrounding the proximal 150bp from each breakpoint.
| Dystrophin Mutation exon(s) involved | Patient code | Breakpoint coordinates a | Distance from upstream exon (bp) b | Distance from downstream exon (bp) c | Deletion Size (bp) | Repetitive elements in proximal breakpoint d | Repetitive elements in distal breakpoint d |
| --- | --- | --- | --- | --- | --- | --- | --- |
| 52 | 101-025 | chrX:g.31711135\_31747638 | 26322 | 31549 | 36503 | none | none |
| | 101-016 | chrX:g.31705631\_31753170 | 20790 | 26045 | 47539 | none | LTR/ERVL |
| | 101-024 | chrX:g.31689664\_31751257 | 22703 | 10078 | 61593 | LINE/L1 and SINE/MIR | LINE/L1 brkp |
| 50-52 | 101-011 | chrX:g.31690355\_31827603 | 9115 | 10769 | 137248 | LINE/L1 | LINE/L1 |
| | 101-022 | chrX:g.31695128\_31825574 | 11144 | 15542 | 130446 | none | LINE/L1 brkp |
| | 101-010 | chrX:g.31721637\_31830759 | 5959 | 42051 | 109122 | 2 LTR/ERV1 | LTR/ERV1 and 2 SR |
| | 101-017 | chrX:g.31700804\_31834364 | 2354 | 21218 | 133560 | SINE/Alu | none |
| 49-52 | 101-015 | chrX:g.31711204\_31869463 | 5725 | 31618 | 158259 | LTR/ERV1 | none |
| | 101-001 | chrX:g.31721941\_31844249 | 30939 | 42355 | 122308 | LINE/L1 | 1 SR and DNA/PiggyBac |
| | 101-012 | chrX:g.31714062\_31873065 | 2123 | 34476 | 159003 | none | none |
| | 101-005 | chrX:g.31715245\_31858865 | 16323 | 35659 | 143620 | none | none |
| | 101-021 | chrX:g.31726548\_31864035 | 11153 | 46962 | 137487 | DNA/hAT-Charlie | none |
| 48-52 | 101-009 | chrX:g.31702738\_31878654 | 50942 | 23152 | 175916 | LINE/L1 | LINE/L2 and DNA/hAT-Charlie |
| | 101-020 | chrX:g.31717393\_31911335 | 18261 | 37807 | 193942 | SINE/MIR and DNA/TcMar-Mariner | none |
| | 101-006 | chrX:g.31682390\_31900411 | 29185 | 2804 | 218021 | LTR/ERVL-MaLR | LTR |
| | 101-023 | chrX:g.31717673\_31886941 | 42655 | 38087 | 169268 | SINE/MIR | none |
| | 101-007 | chrX:g.31699362\_31891377 | 38219 | 19776 | 192015 | DNA/hAT-Charlie | SINE/MIR |
| 45-52 | 101-013 | chrX:g.31729062\_31987098 | 229818 | 49476 | 258036 | LINE/L1 | none |
| | 101-008 | chrX:g.31716321\_32132269 | 84647 | 36735 | 415948 | none | SINE/Alu brkp |
| | 101-003 | chrX:g.31693355\_32099798 | 117118 | 13769 | 406443 | LINE/L1 | 2 LINE/L1 |
| | 101-019 | chrX:g.31691307\_32027577 | 189339 | 11721 | 336270 | none | 2 LINE/L1 |
| | 101-002 | chrX:g.31714273\_32088131 | 128785 | 34687 | 373858 | SINE/MIR | none |
| | 101-014 | chrX:g.31692209\_32161599 | 55317 | 12623 | 469390 | none | 2 LINE/L1 |
| | 101-018 | chrX:g.31715239\_31970340 | 246576 | 35653 | 255101 | none | none |
| | 101-004 | chrX:g.31729058\_32184351 | 32565 | 49472 | 455293 | DNA/hAT-Charlie | none |

## Slide 6
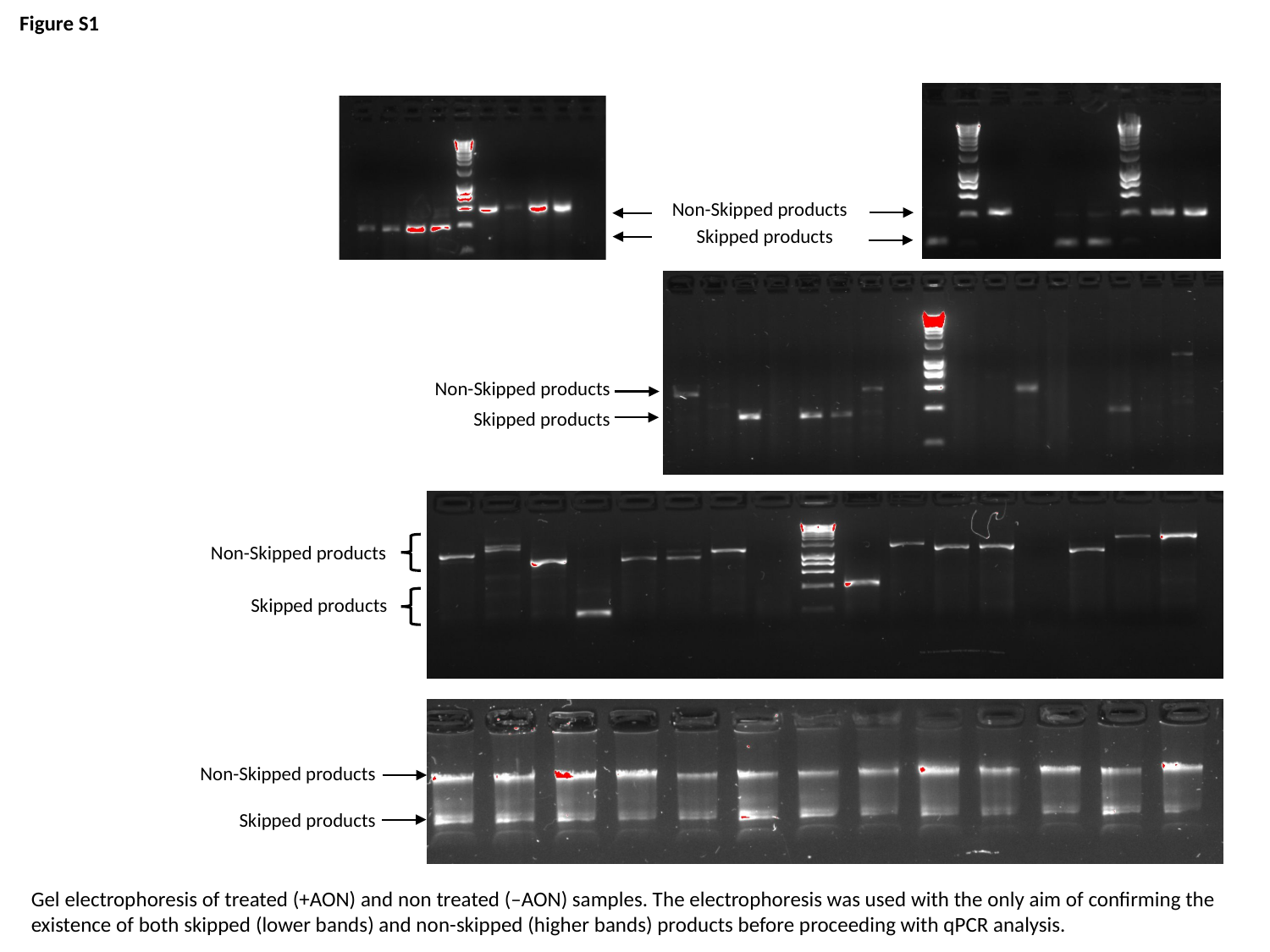

Figure S1
Non-Skipped products
Skipped products
Non-Skipped products
Skipped products
Non-Skipped products
Skipped products
Non-Skipped products
Skipped products
Gel electrophoresis of treated (+AON) and non treated (–AON) samples. The electrophoresis was used with the only aim of confirming the existence of both skipped (lower bands) and non-skipped (higher bands) products before proceeding with qPCR analysis.

## Slide 7
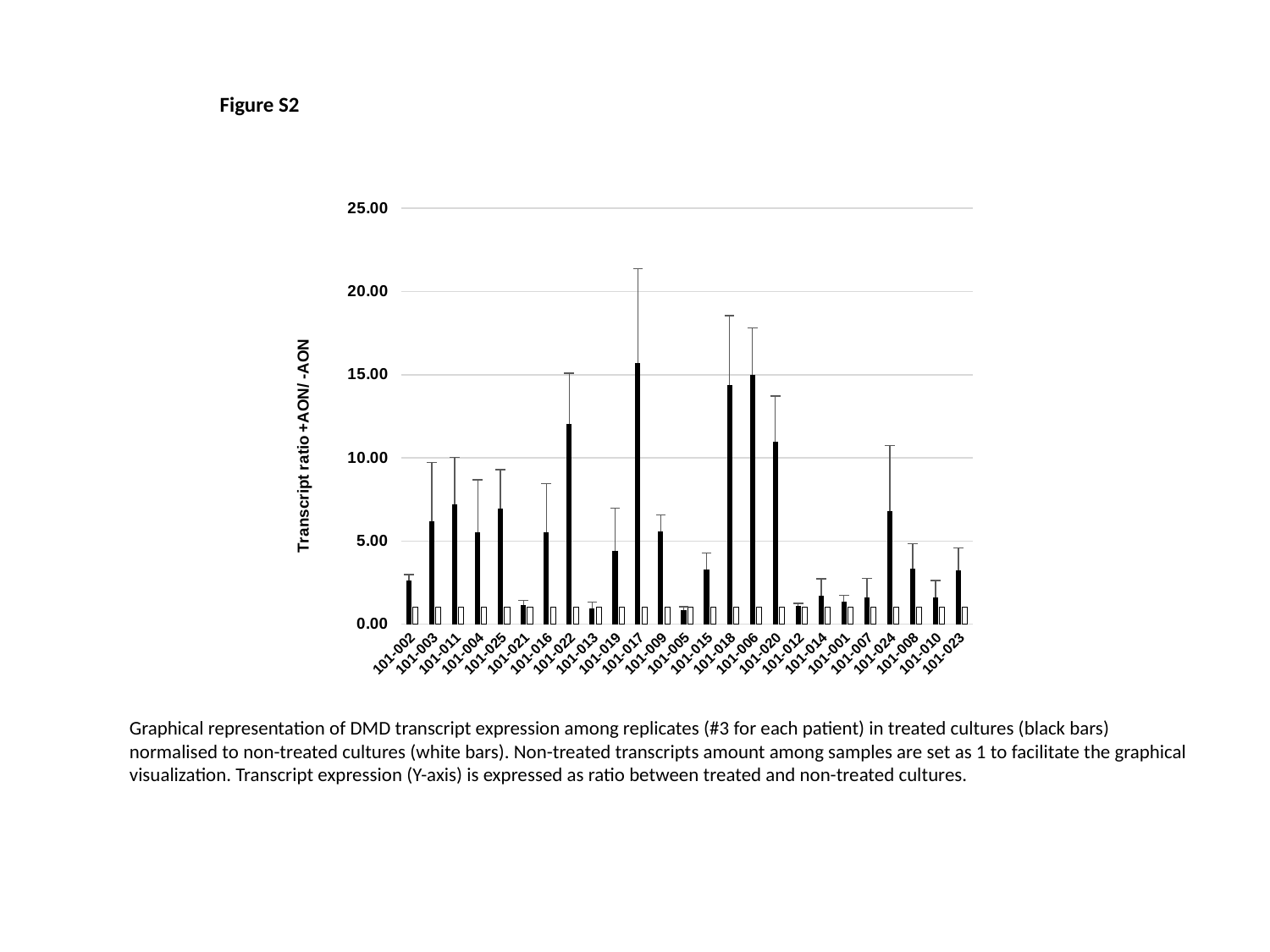

Figure S2
### Chart
| Category | | |
|---|---|---|
| 101-002 | 2.608973754124461 | 1.0 |
| 101-003 | 6.164486345253948 | 1.0 |
| 101-011 | 7.1817100381504115 | 1.0 |
| 101-004 | 5.539850466820851 | 1.0 |
| 101-025 | 6.9361929802567595 | 1.0 |
| 101-021 | 1.142117283970288 | 1.0 |
| 101-016 | 5.514785407675119 | 1.0 |
| 101-022 | 12.031659164556377 | 1.0 |
| 101-013 | 0.946177774047997 | 1.0 |
| 101-019 | 4.420538912425367 | 1.0 |
| 101-017 | 15.713100684653739 | 1.0 |
| 101-009 | 5.585431880954531 | 1.0 |
| 101-005 | 0.8215235399795046 | 1.0 |
| 101-015 | 3.2755170009003773 | 1.0 |
| 101-018 | 14.379217526592383 | 1.0 |
| 101-006 | 14.976739188887956 | 1.0 |
| 101-020 | 10.987216318718064 | 1.0 |
| 101-012 | 1.1088599126836975 | 1.0 |
| 101-014 | 1.7063407686457188 | 1.0 |
| 101-001 | 1.3634418282381382 | 1.0 |
| 101-007 | 1.600952562469286 | 1.0 |
| 101-024 | 6.807797582868138 | 1.0 |
| 101-008 | 3.314539440952131 | 1.0 |
| 101-010 | 1.5902896243722762 | 1.0 |
| 101-023 | 3.240042134558326 | 1.0 |Graphical representation of DMD transcript expression among replicates (#3 for each patient) in treated cultures (black bars) normalised to non-treated cultures (white bars). Non-treated transcripts amount among samples are set as 1 to facilitate the graphical visualization. Transcript expression (Y-axis) is expressed as ratio between treated and non-treated cultures.

## Slide 8
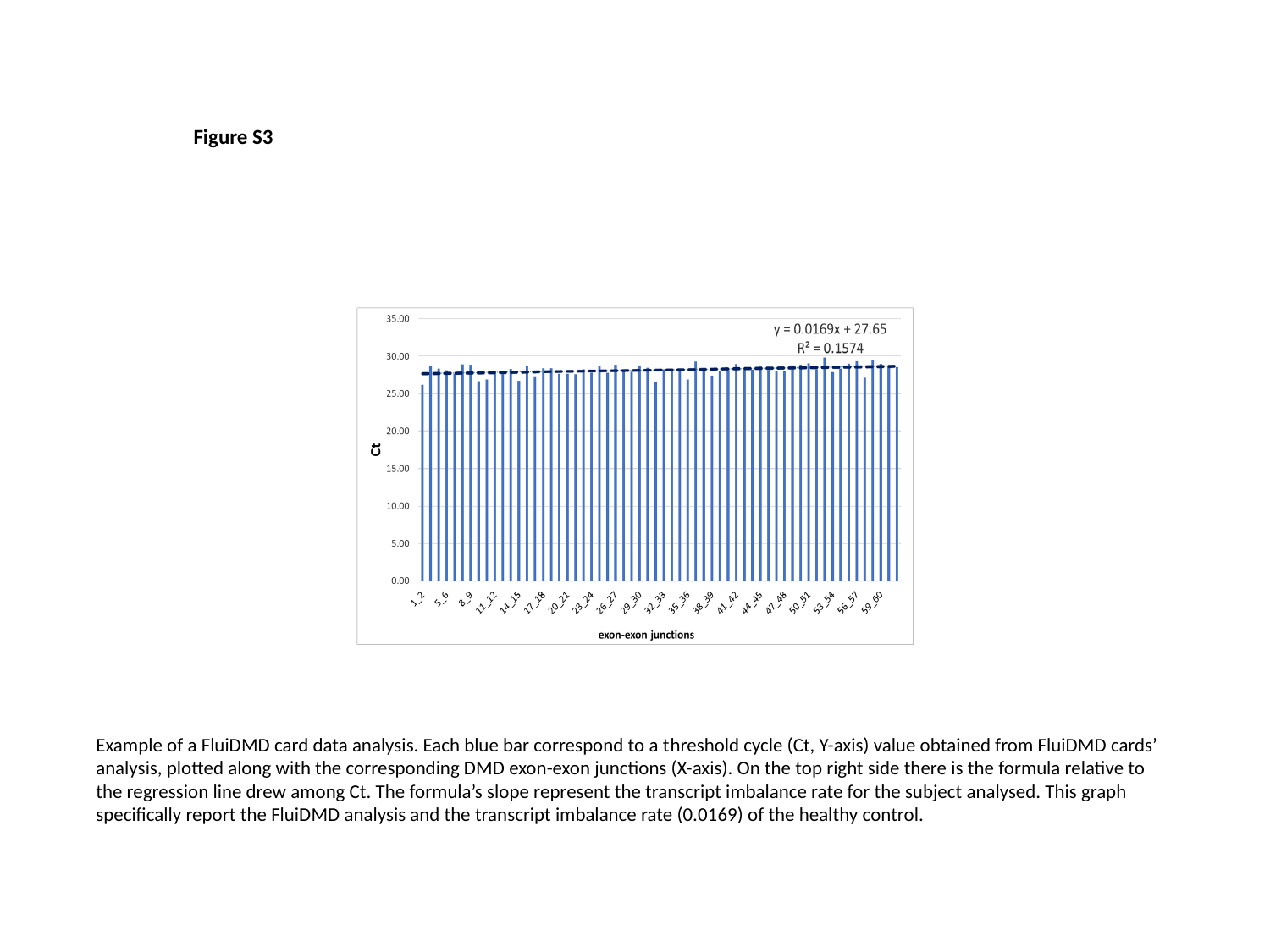

Figure S3
Example of a FluiDMD card data analysis. Each blue bar correspond to a threshold cycle (Ct, Y-axis) value obtained from FluiDMD cards’ analysis, plotted along with the corresponding DMD exon-exon junctions (X-axis). On the top right side there is the formula relative to the regression line drew among Ct. The formula’s slope represent the transcript imbalance rate for the subject analysed. This graph specifically report the FluiDMD analysis and the transcript imbalance rate (0.0169) of the healthy control.

## Slide 9
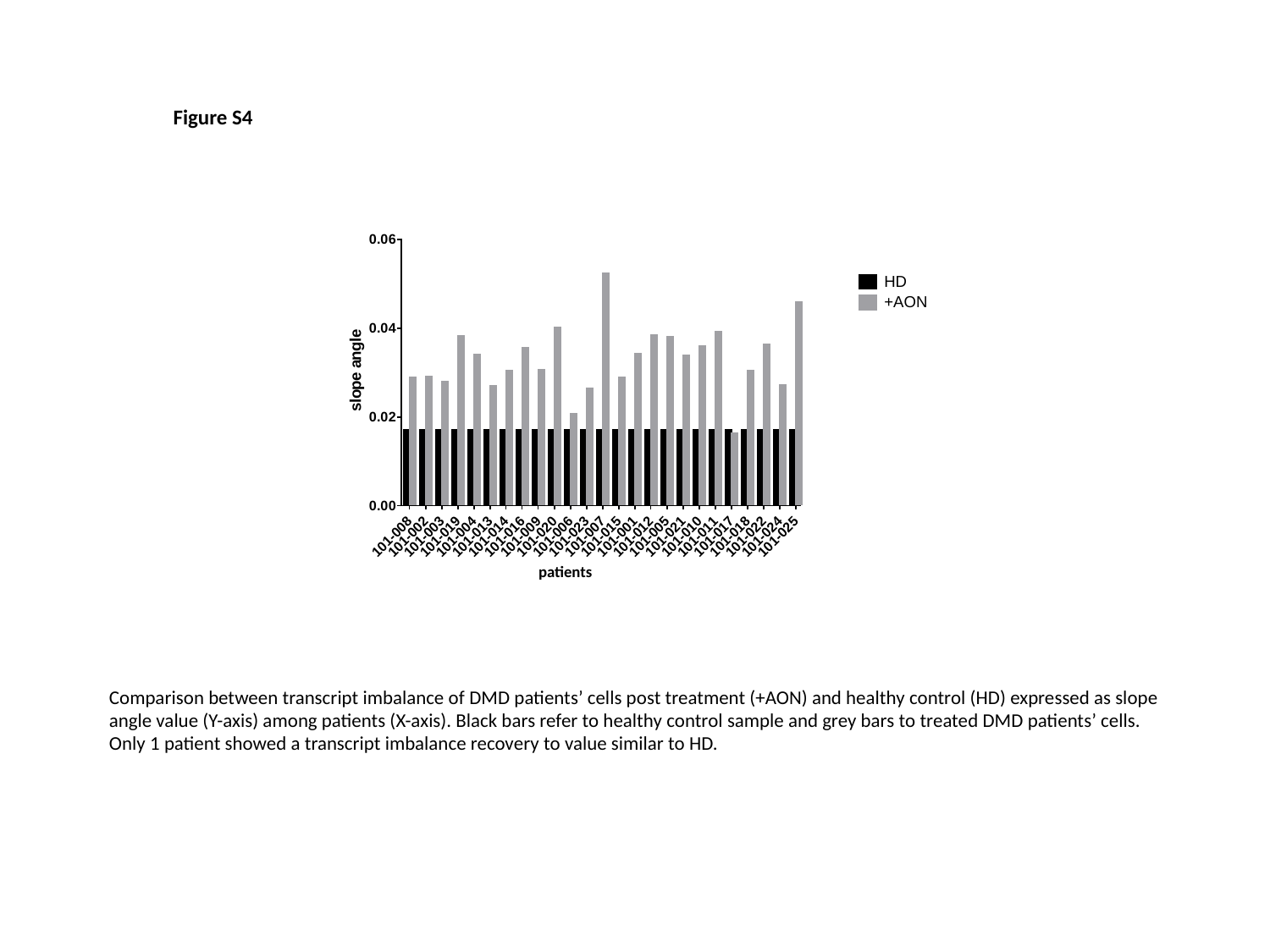

Figure S4
patients
Comparison between transcript imbalance of DMD patients’ cells post treatment (+AON) and healthy control (HD) expressed as slope angle value (Y-axis) among patients (X-axis). Black bars refer to healthy control sample and grey bars to treated DMD patients’ cells. Only 1 patient showed a transcript imbalance recovery to value similar to HD.

## Slide 10
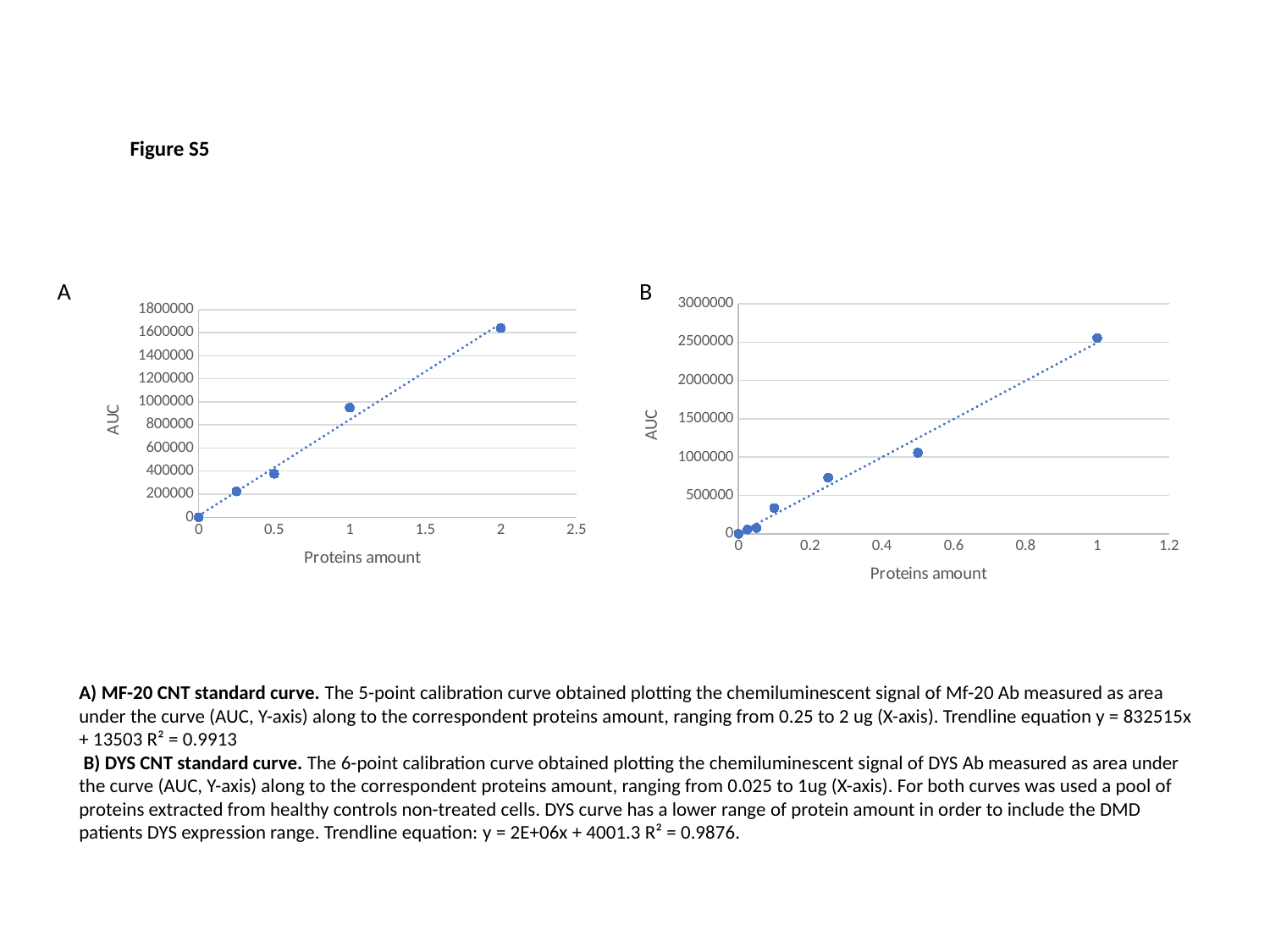

# Figure S5
A
B
### Chart
| Category | |
|---|---|
### Chart
| Category | Average |
|---|---|A) MF-20 CNT standard curve. The 5-point calibration curve obtained plotting the chemiluminescent signal of Mf-20 Ab measured as area under the curve (AUC, Y-axis) along to the correspondent proteins amount, ranging from 0.25 to 2 ug (X-axis). Trendline equation y = 832515x + 13503 R² = 0.9913
 B) DYS CNT standard curve. The 6-point calibration curve obtained plotting the chemiluminescent signal of DYS Ab measured as area under the curve (AUC, Y-axis) along to the correspondent proteins amount, ranging from 0.025 to 1ug (X-axis). For both curves was used a pool of proteins extracted from healthy controls non-treated cells. DYS curve has a lower range of protein amount in order to include the DMD patients DYS expression range. Trendline equation: y = 2E+06x + 4001.3 R² = 0.9876.

## Slide 11
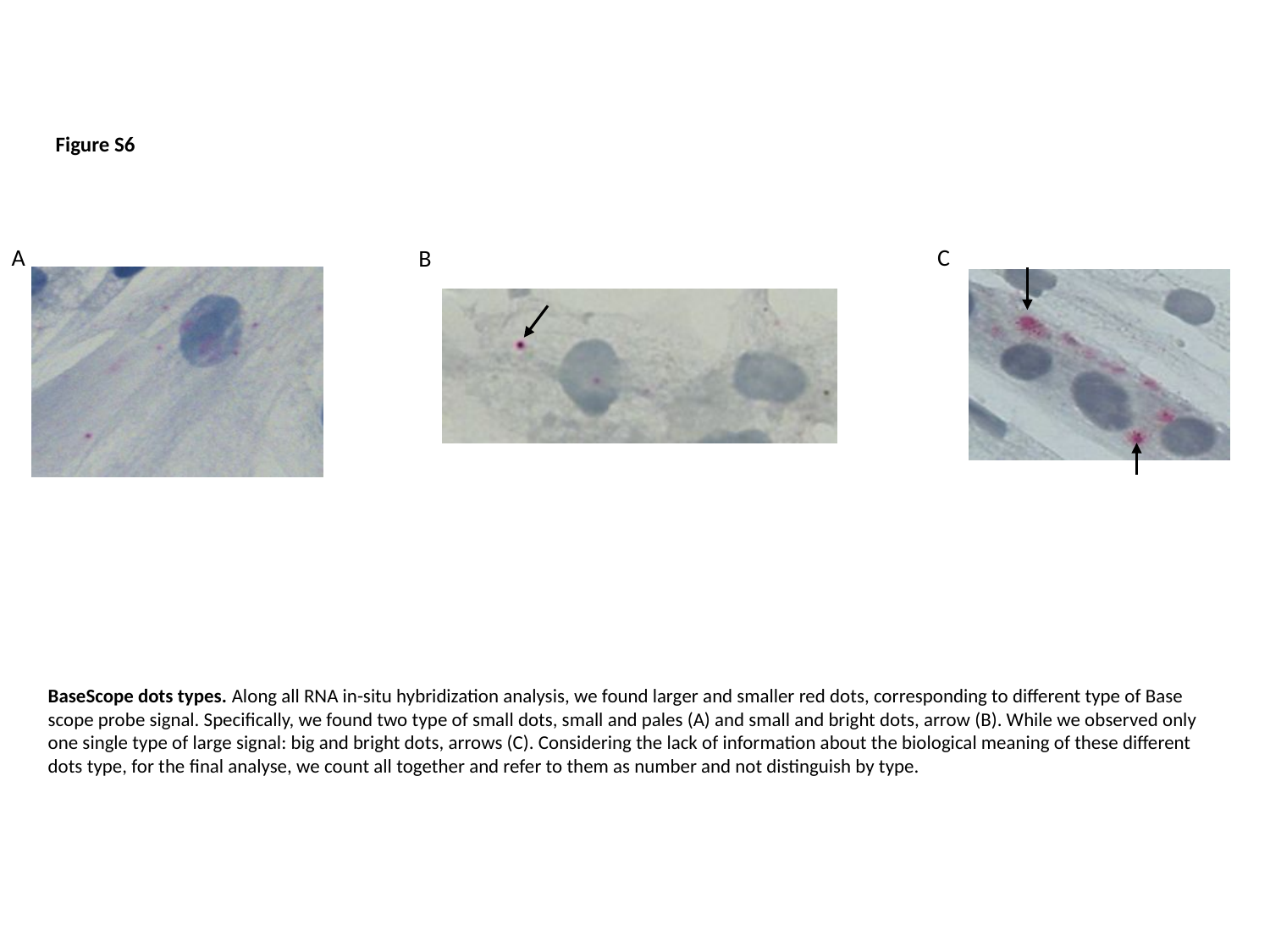

Figure S6
A
C
B
BaseScope dots types. Along all RNA in-situ hybridization analysis, we found larger and smaller red dots, corresponding to different type of Base scope probe signal. Specifically, we found two type of small dots, small and pales (A) and small and bright dots, arrow (B). While we observed only one single type of large signal: big and bright dots, arrows (C). Considering the lack of information about the biological meaning of these different dots type, for the final analyse, we count all together and refer to them as number and not distinguish by type.
